# Dynamic Exposure-Adaptive Learning for Multi-Exposure Image Fusion Using RAW-Derived Training Pairs

**DOI:** 10.3390/s26154855

**Published:** 2026-08-01

**Authors:** Seung Hwan Lee, Sung Hak Lee

**Affiliations:** School of Electronic and Electrical Engineering, Kyungpook National University, 80 Daehakro, Buk-Gu, Daegu 41566, Republic of Korea; hyo98120@knu.ac.kr

**Keywords:** high dynamic range, dynamic range expansion, multi-exposure image fusion, 68T45

## Abstract

Multi-exposure image fusion aims to expand the dynamic range of images by combining complementary information from differently exposed inputs. However, existing high dynamic range (HDR) reconstruction and exposure fusion methods often depend on HDR ground truth, tone mapping, or aligned multi-exposure data. To address these limitations, this study proposes a self-supervised high-exposure (HE) and low-exposure (LE) fusion framework for dynamic range expansion without requiring HDR ground truth. First, RAW-based exposure-range sampling augmentation generates diverse HE-LE training pairs from a single RAW image, expanding the training dataset while providing pixel-level aligned pairs without multi-shot acquisition. Second, a dynamic adaptive fusion module exploits complementary exposure information and balances local detail information with global structural information according to regional exposure characteristics. Fusion quality is progressively improved through Structural Similarity Index Measure (SSIM)-based coarse training and a stage-wise loss optimization strategy. Third, during inference, HDR-like enhancement and color compensation are applied to the LE image before fusion with the HE image to improve luminance, color consistency, and structural detail. Experimental results demonstrate that the proposed framework achieves stable dynamic range expansion without HDR ground truth and outperforms existing methods in structural preservation and visual quality, achieving the lowest BRISQUE (21.214), PIQE (33.979), and SSEQ (20.367) scores, as well as the highest MANIQA score (0.528), among all compared methods.

## 1. Introduction

In digital imaging, dynamic range refers to the range between the darkest and brightest intensity levels that can be captured or represented while preserving distinguishable image details [1,2,3]. When the dynamic range of a scene exceeds the capture or display capability of an imaging system, underexposed regions may lose shadow details, whereas overexposed regions may suffer from highlight saturation. The optical signals captured by camera sensors and reproduced on display devices can differ significantly from human perception, even when representing the same scene at the same moment. These discrepancies arise from limitations in sensor dynamic range, exposure settings, image processing pipelines, and display characteristics. Although advanced sensors and high-performance displays can theoretically achieve or exceed human visual capabilities, their practical adoption is limited by cost and technical constraints. Consequently, HDR imaging research maximizes the available dynamic range within sensor and display limitations while preserving natural appearance and fine image details [4,5].

Recovering HDR content from a single-exposure image remains a challenging task because information lost through saturation or limited dynamic range cannot be physically restored [6,7]. Although inference-based methods can estimate missing content, they often introduce visually unnatural artifacts. Nevertheless, single-image HDR reconstruction has been widely studied due to its data acquisition simplicity and practical scalability [8,9]. However, excessive recovery of lost highlight and shadow details can introduce visual distortions that are not always captured by conventional quantitative metrics.

As an alternative, multi-exposure HDR reconstruction and exposure fusion techniques have been widely investigated [10,11,12]. Multi-exposure image fusion combines multiple images captured under different exposure settings to generate a single image with improved visibility and detail preservation. By combining images captured at different exposures, these methods can recover a wider dynamic range and richer details than single-exposure approaches. However, they face two fundamental challenges. Sequential acquisition with a single camera introduces temporal inconsistencies caused by object motion or illumination changes, whereas simultaneous capture with multiple cameras may produce geometric misalignments and registration errors due to viewpoint differences. Such misalignments can negatively affect the generalization performance of learning-based models, particularly during large-scale dataset construction and training.

The preservation of highlight regions and restoration of shadow details involve a trade-off in fusing HE and LE images. In this study, HE images refer to images captured or generated with relatively high exposure, which generally preserve shadow information but may suffer from highlight saturation. Conversely, LE images refer to images captured or generated with relatively low exposure, which generally preserve highlight details but may lose information in dark regions. Global tone adjustment methods often enhance one region at the expense of another [5,13], while local methods rely heavily on accurate region decomposition and weighting strategies [14,15]. Conventional exposure fusion approaches use luminance-based weighting maps to determine each image’s contribution, but these enforced fusion schemes can introduce unnatural boundaries and artifacts. Therefore, the goal of exposure fusion extends beyond information integration to achieving improved visual quality while preserving image details and natural appearance.

To address the limitations of Multi-exposure Image Fusion, this paper presents the following contributions.

First, a self-supervised HE-LE fusion framework that eliminates the need for HDR ground truth is proposed. Unlike conventional HDR-based methods, which depend on HDR generation, tone mapping, and target construction and are sensitive to the chosen tone-mapping strategy, the proposed framework directly learns HE-LE image fusion without requiring HDR ground truth.

Second, a RAW-based exposure-range sampling augmentation strategy is introduced to generate virtual HE-LE pairs from 14-bit RAW data. By sampling different exposure ranges from a single RAW image, perfectly aligned HE-LE pairs can be created, enabling dataset expansion without additional image acquisition. This allows the network to be trained using large-scale HE-LE pairs with zero alignment error at the training-data level, without relying on multi-shot acquisition for paired exposure data.

Third, a dynamic exposure-adaptive fusion learning strategy is developed to model the complementary characteristics of HE and LE images. To support this learning process, the network employs a feature extraction architecture that separates local detail representation and global exposure-aware representation. In particular, convolution-based feature extraction is used to preserve local structures and textures, while a transformer-based branch is incorporated to capture long-range contextual relationships and global exposure-dependent information. By exploiting the observation that exposure dominance varies across image regions, the network is progressively guided to adaptively utilize information from different exposure levels based on exposure distribution and local image content. To mitigate the trade-off between highlight preservation and shadow restoration, a stage-wise loss optimization is employed, beginning with SSIM-based structural stabilization, followed by progressive enhancement of contrast and dynamic range, and finally refinement of fine details and sharpness. This progressive optimization enables effective learning of cross-exposure complementarity and improves fusion quality.

Finally, during inference, an HDR-like enhancement module and a color compensation process are incorporated as separate components before the final HE-LE fusion. The HDR-like enhancement module is adapted from our previous visible-image enhancement framework. It first generates a synthetic NIR-like image from the visible LE image by emulating the tonal characteristics of NIR imaging, and then fuses the synthetic NIR-like image with the original visible LE image to achieve HDR-like tone redistribution. Separately, since this enhancement process often causes color desaturation and color inconsistency due to significant luminance redistribution, a luminance-variation-based color compensation process is applied to compensate for the inherent color fading problem and preserve color consistency. The resulting color-compensated enhanced LE image is then fused with the original HE image, yielding balanced improvements in luminance, color fidelity, and structural detail.

Overall, this study presents a self-supervised framework for stable and natural HE-LE fusion without requiring HDR ground truth. By eliminating alignment issues through RAW-based data generation and employing a dynamically adaptive fusion strategy, the proposed framework effectively overcomes key limitations of existing exposure fusion methods. Overall, this study presents a self-supervised framework for stable and natural HE-LE fusion without requiring HDR ground truth. By generating pixel-level aligned training pairs through RAW-based data generation and employing a dynamically adaptive fusion strategy, the proposed framework effectively addresses key limitations of existing exposure fusion methods.

The remainder of this paper is organized as follows. Section 2 introduces the background of image enhancement and multi-exposure image fusion. Specifically, Section 2.1 reviews single-image-based enhancement methods, and Section 2.2 discusses multi-exposure image fusion, including traditional methods and deep learning-based methods. Section 3 describes the proposed method. Section 3.1 presents RAW-based exposure-range sampling for HE–LE pair generation, Section 3.2 explains dynamic exposure-adaptive fusion learning, and Section 3.3 describes inference with HDR-like enhancement and color compensation. Section 4 presents the experimental results, including the ablation study, qualitative evaluation, and quantitative evaluation. Finally, Section 5 concludes the paper.

## 2. Background

The problem of expanding scene visibility and information representation using images captured at different exposure levels has been widely studied in image processing. The limited dynamic range of a single-exposure image often causes information loss in dark regions and detail degradation in saturated bright regions. To address these limitations, numerous methods have been developed to integrate complementary information from multiple exposures or generate enhanced visual representations from limited input data.

Existing approaches can be broadly classified into single-image and multi-exposure methods based on the available input information. Single-image methods extend the representation range from a single input without requiring additional images, but their restoration capability is limited by the information contained in that image. In contrast, multi-exposure methods combine complementary information from images captured at different exposure levels to achieve richer visual representations. However, their practical use is hindered by image alignment challenges and the difficulty of constructing well-matched training datasets, which can degrade performance and limit the generalization of deep learning-based models.

From this perspective, this section reviews the relevant literature on dynamic range enhancement and image fusion. First, single-image methods for dynamic range expansion and image enhancement are introduced. Subsequently, Multi-exposure Image Fusion methods are examined, with a focus on both traditional and deep learning-based approaches. Rather than simply listing previous studies, this section analyzes their advantages and limitations in relation to the need for aligned training data, HDR supervision, and exposure-adaptive fusion.

### 2.1. Single Image-Based Enhancement

Single-image enhancement methods have been widely studied because they improve visual quality using only a single input image. Their primary objective is to extend the limited dynamic range of an image and enhance visibility in low-light regions.

Traditional image enhancement methods primarily improve visibility through contrast and dynamic range compression. Jobson et al. [16] proposed Multiscale Retinex (MSR), which combines dynamic range compression with local contrast enhancement based on human visual perception, and later extended it to MSR with color restoration (MSRCR). However, its performance is limited when the gray-world assumption is violated, often causing color distortion. To address this issue, Rahman et al. [17] reformulated the Retinex framework as a nonlinear transformation for automatic image enhancement and further improved color restoration and perceptual quality. Histogram-based methods have also been widely adopted. Zuiderveld et al. [18] proposed contrast-limited adaptive histogram equalization (CLAHE), which adaptively enhances local contrast while suppressing excessive noise amplification through histogram clipping.

More recently, deep learning-based methods have enabled data-driven dynamic range reconstruction and brightness enhancement. Eilertsen et al. proposed an autoencoder-based framework that reconstructs HDR images from a single low dynamic range (LDR) image by recovering information lost in saturated regions, with particular emphasis on restoring luminance and structural details in highlights [6]. Subsequent studies improved reconstruction performance through multi-scale feature learning. ExpandNet introduced a multi-scale architecture that jointly exploits local, medium-scale, and global information to generate HDR images from LDR inputs, achieving more robust dynamic range expansion [19]. Efforts have also been directed toward reducing reliance on training data. Zero-DCE reformulated low-light enhancement as an image-specific curve estimation problem and employed non-reference loss functions, enabling effective brightness and contrast enhancement without paired or unpaired training data [20]. To further overcome the limitations of single-image enhancement, recent studies have incorporated auxiliary representations. In particular, synthetic NIR images generated from visible-light images have been utilized to complement structural and luminance information, enabling HDR-like image reconstruction by exploiting the distinct characteristics of different spectral domains [21].

However, single-image methods are limited by the information contained in the input image and therefore cannot reliably recover details that were not captured. This limitation is particularly pronounced in severely underexposed or overexposed regions, where substantial information loss makes accurate reconstruction challenging.

### 2.2. Multi-Exposure Image Fusion

Multi-exposure Image Fusion generates an enhanced image by integrating complementary information from images captured at different exposure levels. Since a single image can represent only a limited dynamic range, low-exposure images preserve details in bright regions but lose information in dark areas, whereas HE images retain shadow details but often suffer from saturation in highlights. By combining these complementary characteristics, multi-exposure methods aim to achieve a wider dynamic range and improved visual quality beyond what is possible with a single image.

Multi-exposure data are typically acquired either by sequentially capturing images with different exposure settings using a single camera or by simultaneously capturing the scene with multiple cameras. Sequential acquisition inevitably introduces temporal differences between exposures, leading to scene inconsistencies caused by object motion. Although multi-camera systems avoid temporal misalignment, geometric discrepancies and registration errors can still arise from differences in camera positions and viewpoints. Consequently, multi-exposure methods are vulnerable to alignment errors and ghost artifacts originating during data acquisition. In learning-based Multi-exposure Image Fusion systems, such errors not only degrade fusion quality but also provide inconsistent training samples, which may adversely affect model convergence and generalization performance.

Early Multi-exposure Image Fusion studies primarily relied on traditional image processing techniques, such as exposure weighting, pyramid decomposition, and guided filtering, to combine information from multiple images. More recently, convolutional neural network-based approaches have been developed to learn the extraction and fusion of structural features and fine details across exposures. However, traditional methods depend on manually designed rules, limiting their adaptability to complex scenes and diverse imaging conditions. Deep learning-based methods, while more flexible, remain highly dependent on dataset quality, image alignment, and registration accuracy. These limitations indicate that Multi-exposure Image Fusion performance is not determined only by the fusion architecture, but also by how reliably exposure-complementary training pairs are constructed and how adaptively exposure-dependent information is utilized. Accordingly, the following subsections review traditional and deep learning-based Multi-exposure Image Fusion methods.

#### 2.2.1. Traditional Methods

Traditional Multi-exposure Image Fusion methods aim to integrate complementary information from images captured at different exposure conditions. By combining structural and luminance information through techniques such as contrast-, saturation-, and well-exposedness-based weighting, pyramid decomposition, and structural decomposition, these methods seek to produce visually natural images with an extended dynamic range.

Goshtasby et al. [22] proposed a Multi-exposure Image Fusion method that selects local regions with the highest information content using entropy and smoothly combines them through Gaussian blending. The block size and blending parameters were further optimized via entropy maximization to enhance the information content of the fused image.

Subsequently, Mertens et al. [11] introduced an exposure fusion method that directly combines multiple exposure images without explicit HDR reconstruction. Their approach employs contrast-, saturation-, and well-exposedness-based weighting maps together with Laplacian and Gaussian pyramid-based multi-resolution blending to produce visually natural fusion results.

Subsequent studies further advanced Multi-exposure Image Fusion by enhancing structural information utilization and reducing fusion artifacts. Ma et al. [23] proposed a structural patch decomposition-based fusion method that separately decomposes image patches into signal strength, signal structure, and mean intensity components before fusing them independently. Structural consistency was also exploited to suppress ghost artifacts effectively without requiring explicit motion estimation.

Hayat et al. [24] proposed a ghost-free Multi-exposure Image Fusion method that extracts local contrast features using dense SIFT descriptors and refines weighting maps through guided filtering. By jointly exploiting local contrast, brightness, and color dissimilarity information, the method effectively suppresses ghost artifacts in dynamic scenes.

Subsequently, Li et al. [25] extended structural patch decomposition-based Multi-exposure Image Fusion into a multi-scale framework to improve computational efficiency and reduce halo artifacts. By integrating structural information across multiple scales, the method effectively suppresses both ghost and halo artifacts in dynamic scenes.

However, most traditional Multi-exposure Image Fusion methods depend on hand-crafted features and fixed fusion rules, limiting their adaptability to complex scenes and varying illumination conditions. They are also highly sensitive to input image misalignment, which can lead to halo artifacts, ghost artifacts, and color inconsistencies depending on the weighting and decomposition strategies employed. These limitations become particularly pronounced in dynamic scenes, where object motion and illumination changes often cause structural discontinuities and detail loss. This indicates that rule-based fusion strategies alone are insufficient to adaptively determine the contribution of HE and LE images across regions with different exposure characteristics.

To address these limitations, recent research has increasingly adopted deep learning-based Multi-exposure Image Fusion methods, which use data-driven learning to model structural relationships and luminance variations across exposure images more effectively.

#### 2.2.2. Deep Learning-Based Methods

Recently, convolutional neural network (CNN)-based methods have been widely explored for data-driven extraction and fusion of structural features and fine details from multi-exposure images. Unlike traditional hand-crafted approaches, these methods can learn complex luminance distributions and structural relationships across images captured under different exposure conditions.

Prabhakar et al. [26] proposed DeepFuse, a CNN-based framework trained with a no-reference MEF-SSIM loss, eliminating the need for ground-truth fused images. The method extracts common low-level features from images with different exposures and integrates them to produce artifact-free fusion results. Subsequently, DenseFuse introduced an encoder–decoder fusion framework that employs convolutional layers and dense blocks to preserve multi-level feature information. In this architecture, deep features extracted by the encoder are adaptively fused and then reconstructed into the final fused image by the decoder [27].

Zhang et al. [28] proposed IFCNN, a general CNN-based image fusion framework applicable to multi-focus, infrared-visible, medical, and Multi-exposure Image Fusion tasks. The method extracts convolutional features from input images, combines them through an element-wise fusion strategy, and reconstructs the fused image using convolutional layers. Subsequent studies emphasized improved preservation of luminance and structural information. PMGI introduced a unified image fusion network that proportionally preserves gradient and intensity information by separating gradient and intensity processing paths. This design enables simultaneous retention of texture details and intensity distributions, while feature reuse and path-wise transfer blocks enhance performance across diverse image fusion tasks [29].

Ma et al. [30] proposed MEF-Net, which combines CNNs with guided filtering for Multi-exposure Image Fusion. The method predicts a low-resolution weight map and employs guided filtering-based joint upsampling, achieving both high fusion quality and computational efficiency. Similarly, Xu et al. [31] introduced U2Fusion, an unsupervised fusion framework that unifies multi-modal, multi-exposure, and multi-focus image fusion within a single architecture. The framework incorporates an adaptive information preservation mechanism, enabling effective learning across diverse fusion tasks without requiring ground-truth fused images.

More recently, transformer-based architectures have been explored to capture long-range dependencies in image fusion tasks. Qu et al. [32] proposed TransMEF, a self-supervised Multi-exposure Image Fusion framework that integrates CNN and transformer architectures to jointly exploit local and global information while learning multi-exposure characteristics without requiring ground-truth fused images.

**Figure 1:**
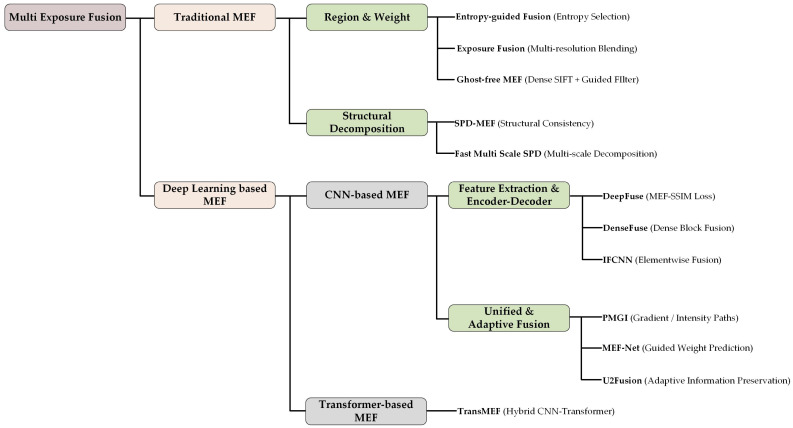
Taxonomy of traditional and deep learning-based Multi-exposure Image Fusion methods.

Figure 1 categorizes Multi-exposure Image Fusion methods into traditional and deep learning-based approaches and illustrates their key characteristics and evolutionary trends. Traditional methods are further divided into region/weight-based and structural decomposition-based approaches, whereas deep learning-based methods show a progression from CNN-based architectures to transformer-based architectures.

However, deep learning-based Multi-exposure Image Fusion methods remain highly dependent on the quality and characteristics of the training data. Multi-exposure images are particularly susceptible to misalignment caused by object motion, camera shake, and exposure variations during acquisition. Such misalignment can propagate through training, leading to ghost artifacts and structural distortions in the fused results. Moreover, supervised methods require ground-truth fused images, whose acquisition in real HDR environments is highly challenging due to alignment constraints. Although unsupervised methods avoid ground-truth supervision, they remain sensitive to structural inconsistencies and exposure distribution variations among input images, often resulting in degraded performance in complex scenes. Therefore, the main limitation of learning-based Multi-exposure Image Fusion is not only the network architecture itself, but also the difficulty of constructing reliable aligned training pairs and defining appropriate supervision without HDR ground truth.

Consequently, there is a growing need for learning-based Multi-exposure Image Fusion methods that can mitigate alignment issues while ensuring robust structural and luminance preservation across diverse exposure conditions. From the above review of Multi-exposure Image Fusion methods, the remaining limitations can be summarized in two aspects. First, traditional Multi-exposure Image Fusion methods do not require HDR ground truth but depend on hand-crafted weighting and decomposition rules, which limits their adaptability to region-dependent exposure characteristics. Second, deep learning-based Multi-exposure Image Fusion methods improve representation capability but remain sensitive to dataset quality, image alignment, and target construction. Existing HDR-based approaches typically depend on linear HDR reconstruction, tone mapping, and target construction processes, making their performance sensitive to the chosen tone-mapping strategy. Furthermore, multi-shot datasets are prone to misalignment caused by object motion and camera shake during acquisition, which can introduce ghost artifacts and structural distortions into the learning process.

Based on this analysis, this study proposes a self-supervised HE-LE fusion framework that eliminates the need for HDR ground truth. Fully aligned HE-LE pairs are generated by sampling different exposure ranges from a single 14-bit RAW image, avoiding the alignment issues in multi-shot acquisition. Using these RAW-derived training pairs, the proposed framework adaptively fuses structural and luminance information according to the exposure characteristics of the input images. Furthermore, an HDR-like enhancement module is incorporated during inference to compensate for luminance and structural deficiencies in underexposed regions, enabling stable fusion performance across diverse exposure conditions.

## 3. Proposed Method

**Figure 2:**
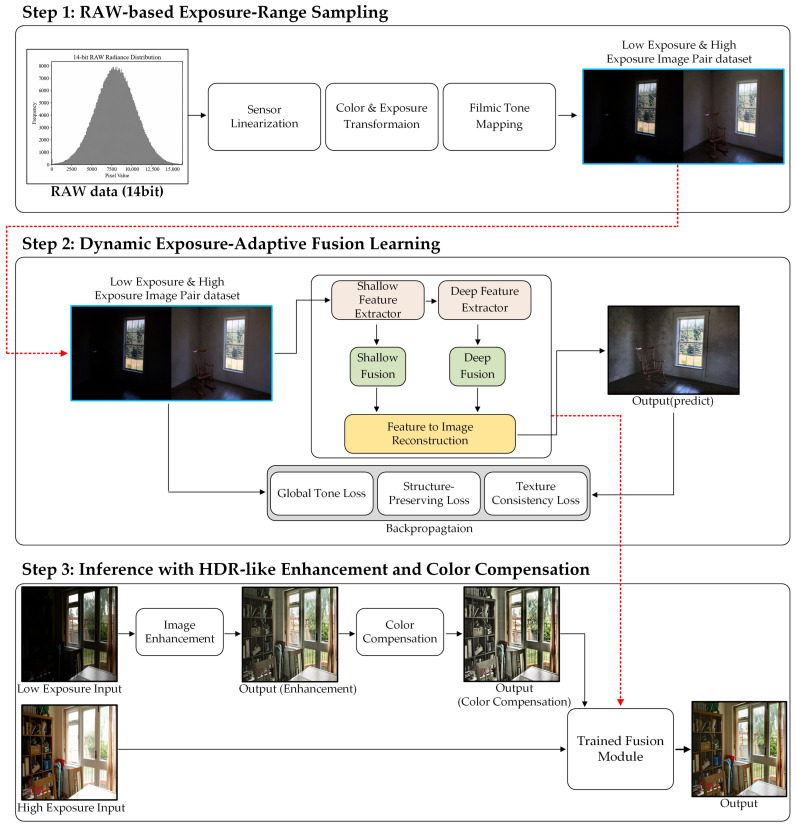
Overview of the proposed framework.

This section presents the overall architecture and key components of the proposed HE-LE fusion framework. The framework comprises three stages: data construction, training, and inference (Figure 2).

First, a RAW-based data generation strategy is introduced to create fully aligned HE-LE pairs by sampling multiple exposure levels from a single 14-bit RAW image. This approach enables the efficient HE-LE dataset construction without additional image acquisition and eliminates the alignment issues associated with multi-shot capture.

Based on the generated HE-LE pairs, an adaptive fusion framework is developed to dynamically regulate information contributions according to the input exposure distribution. Since HE and LE images provide complementary information across different spatial regions rather than merely differing in brightness, the proposed framework is designed to exploit these region-dependent characteristics. This concept is analogous to multi-band image fusion, where complementary information from different modalities is integrated to form a unified representation.

In particular, previous studies, such as MPC-Fusion, have shown that separating shallow and deep features effectively captures complementary information characteristics [33]. Motivated by this observation, the proposed dynamic exposure-adaptive fusion module is designed to adaptively regulate the contributions of local texture and global structural information according to exposure conditions.

Furthermore, a stage-wise loss optimization is introduced to mitigate the trade-off between highlight preservation and shadow restoration. During the initial training stage, the network prioritizes structural stability and global tone consistency. As training progresses, greater emphasis is placed on contrast enhancement and fine-detail restoration, gradually improving fusion quality. Combined with the proposed data construction strategy, this approach enables stable HE-LE fusion learning without HDR ground truth.

Finally, during inference, HDR-like enhancement and luminance variation-based color compensation are applied to the LE image before fusion with the HE image. This process enhances visibility in low-light regions while preserving tonal balance and color consistency, producing visually natural fusion results.

### 3.1. RAW-Based Exposure Range Sampling for HE-LE Pair Generation

A fundamental challenge in Multi-exposure Image Fusion is the acquisition of well-aligned HE-LE image pairs. Images captured under different exposure settings inevitably contain temporal and spatial inconsistencies that can propagate through learning-based models. This issue becomes more critical when constructing large-scale datasets, where perfect alignment is difficult to achieve in practice. To address this limitation, the proposed method avoids multi-exposure acquisition and instead generates 8-bit HE-LE images with different exposure ranges from a single 14-bit RAW image.

RAW data preserves linear sensor measurements and retains a substantially wider dynamic range than conventional 8-bit sRGB images. Consequently, different exposure conditions can be simulated by adjusting the exposure range in the RAW domain, enabling the generation of virtual HE-LE pairs from a single image without additional image acquisition.

**Figure 3:**
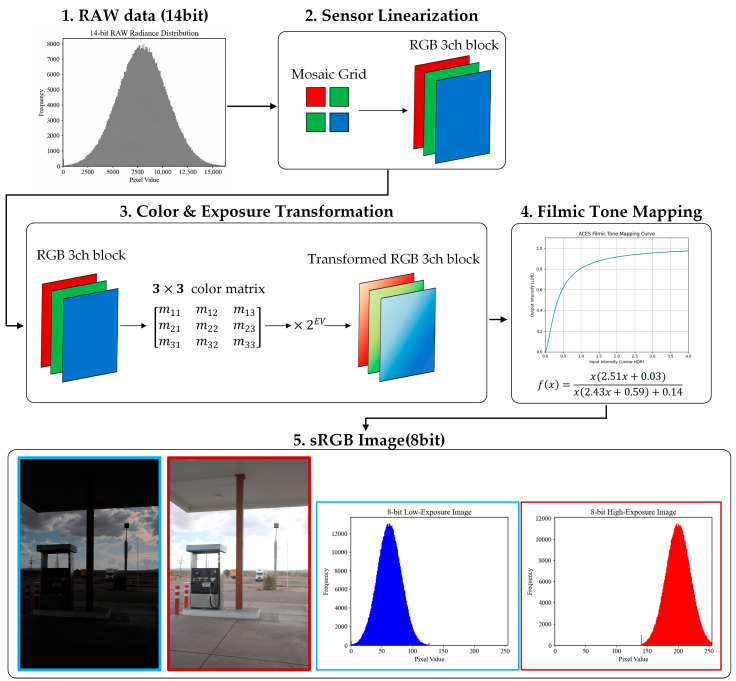
RAW-based HE-LE pair generation process.

Specifically, exposure scaling and tone compression are applied in the RAW domain while preserving linear characteristics of the sensor signal, enabling the generation of 8-bit images with different luminance ranges. Since all HE-LE pairs are derived from a single RAW source, the proposed approach avoids the geometric misalignments and temporal variations associated with multi-shot acquisition. Consequently, it reduces data collection costs while improving training stability and reproducibility. An overview of the HE-LE pair generation process is presented in Figure 3.

Figure 3 illustrates the procedure for generating HE-LE pairs from a 14-bit RAW image. The input RAW data consist of linear sensor measurements stored in a Bayer mosaic pattern. The RAW signal is then reconstructed into a three-channel RGB image through sensor linearization. At this stage, the resulting RGB values remain in the camera’s native color space.

Next, a device-specific 3 × 3 color transformation matrix is applied to convert the camera-dependent RGB data into a standard color space while preserving the linear characteristics of the RAW signal, producing linear 14-bit RGB data.

Subsequently, Filmic tone mapping compresses the linear 14-bit RGB signal into the perceptually meaningful 8-bit sRGB domain. By providing smooth highlight roll-off and stable preservation, Filmic tone mapping naturally redistributes the linear HDR. This process generates 8-bit images with different exposure ranges.

**Figure 4:**
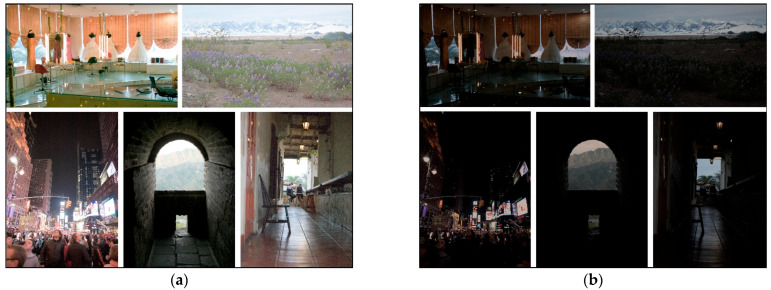
Examples of generated HE–LE image pair: (**a**) High-exposure Images; (**b**) Low-exposure Images.

Finally, LE and HE images are generated by adjusting the tone-mapping parameters to produce different luminance distributions. The corresponding histograms illustrate how the exposure range is redistributed under different settings. Since both images originate from the same 14-bit RAW source, they remain perfectly aligned in both temporal and spatial domains. Consequently, HE-LE datasets with diverse exposure characteristics can be constructed without alignment errors. Figure 4 presents examples of the generated fully aligned HE-LE pairs.

### 3.2. Dynamic Exposure-Adaptive Fusion Learning

Although HE and LE images share identical spatial and temporal content, their contributions to local details and global structural information vary across image regions depending on the exposure conditions. Consequently, no single exposure can be assumed to consistently provide either texture or structural information. Therefore, role-specific fusion strategies commonly used in conventional modality fusion tasks cannot be directly applied to HE-LE fusion.

Furthermore, HE-LE fusion involves a trade-off between luminance enhancement and detail preservation. When the richer shadow information in the HE image is used to recover dark-region details, the corresponding low-illumination content in the LE image may adversely affect the fusion process. Similarly, when the well-preserved highlight information in the LE image is exploited to retain bright-region details, the corresponding saturated regions in the HE image may introduce interference. Consequently, balancing these conflicting exposure characteristics is crucial but difficult to achieve with a single fixed objective function.

**Figure 5:**
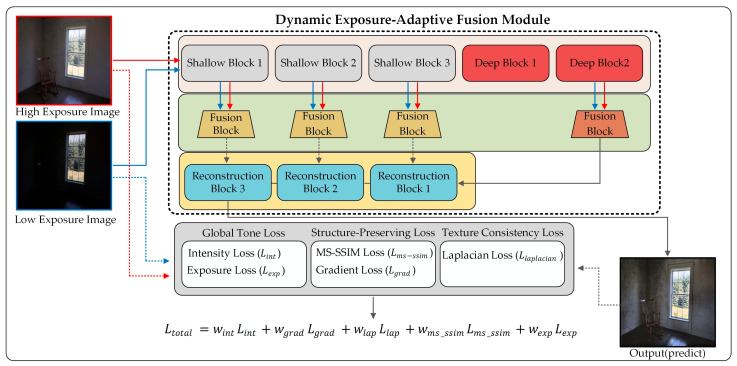
Training pipeline of the proposed fusion framework.

To address these challenges, this study redesigns both the learning framework and optimization objectives while retaining the overall network architecture. First, a dynamic exposure-adaptive fusion module is introduced as a role-independent fusion design that avoids assigning predefined roles to specific exposure levels. Instead, the module learns to adaptively regulate the information contribution of HE and LE features according to the input exposure distribution. The stage-wise loss optimization strategy is then applied to guide this fusion process during training, rather than using a fixed loss function throughout the entire optimization process. The overall framework, illustrated in Figure 5, presents the complete pipeline from data input and feature extraction to fusion and the associated loss functions.

**Figure 6:**
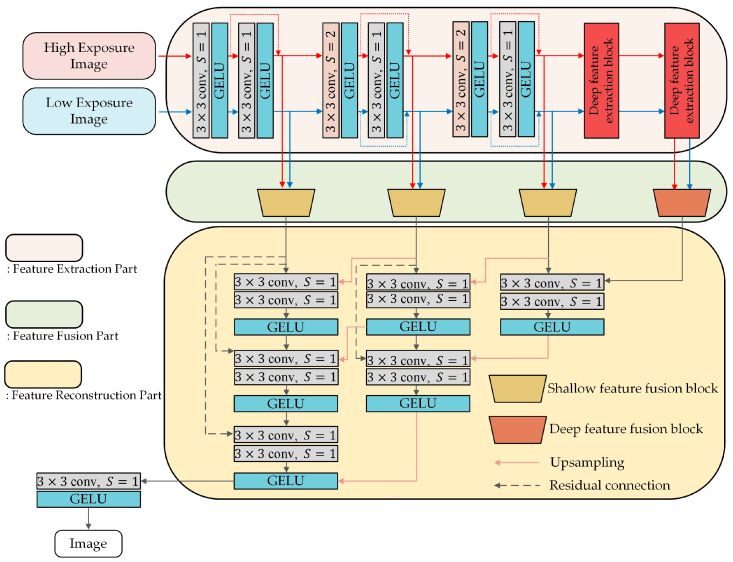
Dynamic exposure-adaptive fusion module’s architecture.

The core component of the proposed framework is the dynamic exposure-adaptive fusion module. Figure 6 presents its detailed architecture. Given the HE and LE inputs, the proposed module processes each image through two parallel paths: a shallow convolutional path and a deep transformer-based path. The shallow path extracts local textures and boundary details, whereas the deep path captures global structural and exposure-aware contextual information. The HE and LE shallow features are then fused by the shallow feature fusion block, and the HE and LE deep features are fused by the deep feature fusion block. Finally, the fused multi-scale features are delivered to the reconstruction module, where high-level fused features are progressively up-sampled and combined with shallow fusion features through skip connections to generate the final fused image.

The shallow feature extractor comprises a basic block containing two consecutive 3 × 3 convolution + GELU operations. Three such blocks are stacked sequentially to progressively enhance local textures and fine details while preserving spatial resolution. In contrast, the deep feature extractor is based on the lightweight transformer encoder architecture of MPC-Fusion [28]. Each block first captures local features through a local perception unit (LPU) and then models global contextual information using spatially reduced multi-head self-attention. Furthermore, the feed-forward network replaces convolutional fully connected layers with a convolutional design comprising 1 × 1 and depth-wise feed-forward network convolutions, improving computational efficiency while preserving spatial information. Consequently, the deep branch effectively captures global structures and long-range dependencies in HE and LE images.

Through this architecture, the network hierarchically captures the complementary characteristics of HE-LE images by extracting local textures and fine details in the shallow branch and global structural and contextual information in the deep branch. The shallow features are integrated through a shallow feature fusion block, which employs attention-based feature recalibration to effectively fuse low-level texture and boundary information from different exposures. Meanwhile, the deep features are combined using a deep feature fusion block, which leverages a cross-attention mechanism to model structural relationships and global contextual interactions between the HE and LE images.

The fused multi-scale features are then passed to the reconstruction module for final image generation. This module adopts an encoder–decoder architecture that progressively up-samples high-level features while integrating them with the corresponding shallow fusion features. Skip connections are also incorporated to preserve low-level details throughout the reconstruction process. Consequently, both local texture and global structural information are effectively utilized, enabling the generation of fused images with natural luminance distributions and strong structural consistency.

Building upon the proposed architecture, the objective functions are redesigned to better accommodate the characteristics of HE-LE fusion. To mitigate the trade-off between different exposure levels, a stage-wise loss optimization strategy is introduced, where the optimization objectives evolve throughout training instead of relying on a single fixed loss function. Specifically, separate loss terms are defined to capture global luminance information, structural fidelity, and fine texture consistency. These losses are selectively combined and adaptively weighted at different training stages, enabling the network to progressively achieve improved fusion performance.

1. Global Structure Stabilization (Stage 1)

During the initial training stage, the network focuses on establishing global structural stability and tonal consistency. To achieve this, intensity-, gradient-, and structural similarity-based losses are jointly optimized. First, a joint intensity representation is constructed by selecting, at each pixel location, the intensity value that contains relatively richer information from either the *HE* or *LE* images:

(1) $$I_{joint}\left(x\right)=max(I_{HE}\left(x\right),I_{LE}(x))$$

where (x) denotes the spatial coordinate, and $I_{HE}\left(x\right)$ and $I_{LE}(x)$ represent *HE* and *LE* input images, respectively. The intensity loss is then defined as

(2) $$L_{int}={\left\|F\left(x\right)-I_{joint}(x)\right\|}_{1}$$

where F(x) denotes the fused image generated by the proposed framework. This loss promotes the preservation of global luminance information. To further maintain structural sharpness, gradient information is incorporated using the Sobel operator:

(3) $$G_{joint}\left(x\right)=max(G_{HE}\left(x\right),G_{LE}(x))$$

where $G_{HE}\left(x\right)$ and $G_{LE}(x)$ represent the spatial gradient information extracted from HE and LE images, respectively. The gradient loss is then defined as

(4) $$L_{grad}={\left\|\nabla F\left(x\right)-G_{joint}(x)\right\|}_{1}$$

This loss helps preserve edge and boundary structures. To further model the spatially varying relationship between *HE* and *LE* images, a texture-guided structural similarity measure is introduced. The low-frequency component of each image is first extracted using a guided filter, and the detail component is then obtained as

(5) $$I_{smooth}=GF\left(I,\gamma ,\epsilon \right),\;\;I_{detail}=I-I_{smooth}$$

where $GF\left(I,\;\gamma ,\;\epsilon \right)$ represents guided filtering, and  γ and  ϵ denote the filter radius and regularization parameters, respectively. The extracted *HE* and *LE* detail components are symmetrically combined to construct a texture reference:

(6) $$T_{GT}\left(x\right)=\left|I_{detail}^{HE}(x)\right|+\left|I_{detail}^{LE}(x)\right|$$

where $I_{detail}^{HE}(x)$ and $I_{detail}^{LE}(x)$ represent the detailed components of the *HE* and *LE* images, respectively. The corresponding detail component of the fused image is similarly extracted to form the predicted representation:

(7) $$T_{pred}\left(x\right)=F\left(x\right)-GF(F\left(x\right),\gamma ,\epsilon )$$

Multi-scale structural similarity is then used to measure fine-detail consistency:

(8) $$L_{SSIM}=1-MS\_SSIM(T_{pred},T_{GT})$$

where $T_{GT}$ and $T_{pred}$ represent the detail-based representations constructed from the input and the fused images, respectively.

The total loss in Stage 1 is defined as

(9) $$L_{total}={\lambda_{int}L}_{int}+\lambda_{grad}L_{grad}+\lambda_{SSIM}L_{SSIM}$$

This formulation promotes stable global luminance distributions and structural consistency.

2. High Frequency Detail Enhancement (Stage 2)

In the second stage, a Laplacian-based loss is introduced to strengthen high-frequency details:

(10) $$L_{lap}={\left\|△F-max(△I_{HE},△I_{LE})\right\|}_{1}$$

where △ denotes the Laplacian operator. By referencing the dominant high-frequency component from the input images, this loss strengthens edge and texture preservation. Consequently, fine details are progressively enhanced while retaining the structural stability achieved in the previous stage.

3. Detail Dominant Transition (Stage 3)

In the third stage, the optimization emphasis transitions from structure-oriented learning to detail-oriented learning. Accordingly, the weights of the SSIM and intensity losses are reduced, while those of the gradient and Laplacian losses are increased. To prevent highlight saturation caused by excessive high-frequency enhancement, a clipping constraint is introduced:

(11) $$L_{clip}=\frac{1}{N}\sum max(F-0.95,\;0)$$

where N represents the total number of pixels. This constraint limits excessive brightness and stabilizes the fused output. Consequently, the optimization focus is gradually shifted from structural preservation to high-frequency detail enhancement.

4. Exposure-adaptive Refinement (Stage 4)

The final stage introduces exposure-adaptive refinement through luminance-dependent weight maps derived from the input images:

(12) $$W_{LE}=\frac{I_{LE}-min(I_{LE})}{\max\left(I_{LE}\right)-min(I_{LE})},\;{\;W}_{HE}=1-W_{LE}$$

Based on the defined weight maps, an exposure-aware reference is formulated as

(13) $$F_{gt}^{exp}=W_{LE}\cdot I_{LE}+W_{HE}\cdot I_{HE}$$

An additional L1 loss is then introduced:

(14) $$L_{exp}={\left\|F-F_{gt}^{exp}\right\|}_{1}$$

In this stage, the clipping constraint is removed to allow exposure-adaptive refinement. The overall loss function is therefore defined as

(15) $$\begin{array}{cc} L_{total}=w_{int}L_{int} & +\;w_{grad}L_{grad}+w_{lap}L_{lap}+w_{ms\_ssim}L_{ms\_ssim}+w_{exp}L_{exp}\\ & +\;w_{clip}L_{clip} \end{array}$$

where the loss weights are adaptively adjusted according to the training stage. Table 1 summarizes the weight settings of the proposed stage-wise loss optimization strategy.

Table 1 summarizes the loss-weight configurations used in the proposed stage-wise loss optimization strategy. In Stage 1, a relatively large weight is assigned to the MS-SSIM loss (30) to prioritize global structural stability and luminance consistency. Rather than emphasizing fine-detail restoration, this stage focuses on a stable fusion foundation through structural preservation and overall tonal alignment.

In Stage 2, the original loss configuration is retained, and a Laplacian loss is introduced to promote high-frequency detail learning. This allows the network to progressively enhance edge and texture information while maintaining the structural stability achieved in Stage 1.

Stage 3 serves as a transition from structure-oriented to detail-oriented optimization. Although relatively short, this stage substantially reduces the MS-SSIM weight from 30 to 5 while doubling the weights of the gradient and Laplacian losses. Consequently, the optimization focus shifts from structural preservation toward fine-detail enhancement. Furthermore, a clipping loss is introduced to suppress highlight saturation caused by aggressive high-frequency enhancement.

Stage 4 introduces an exposure-aware loss while retaining the existing structural and detail-preserving losses. This term guides the network to adaptively utilize the dominant exposure information from either the HE or LE image in each region. Unlike Stage 3, the clipping constraint is removed to allow greater flexibility in luminance representation. Consequently, the final stage refines the fusion output by jointly optimizing structural consistency, fine-detail preservation, and exposure adaptivity.

### 3.3. Inference with HDR-like Enhancement and Color Compensation

**Figure 7:**
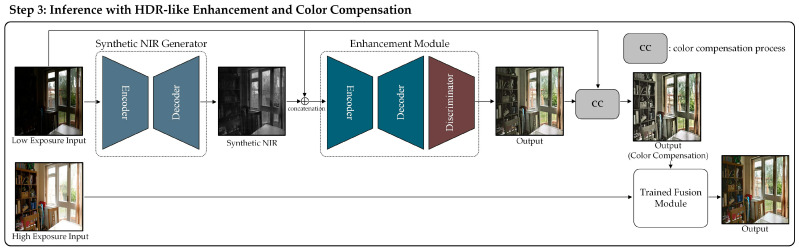
HDR-like enhanced inference and color compensation framework.

During inference, an additional preprocessing stage is applied to enhance the LE image fusion. Specifically, the HDR-like enhancement framework proposed in [21] is employed to adjust the luminance distribution and improve visibility in low-light regions. Originally developed for single-image enhancement, this framework generates a synthetic NIR image from a visible-light image and exploits NIR-like tonal characteristics to extend the effective dynamic range. In the proposed method, it is repurposed as a pre-processing module within the fusion pipeline. Consequently, the enhanced LE image provides more complementary luminance and structural information for fusion with the original HE image. The overall inference process is illustrated in Figure 7.

**Figure 8:**
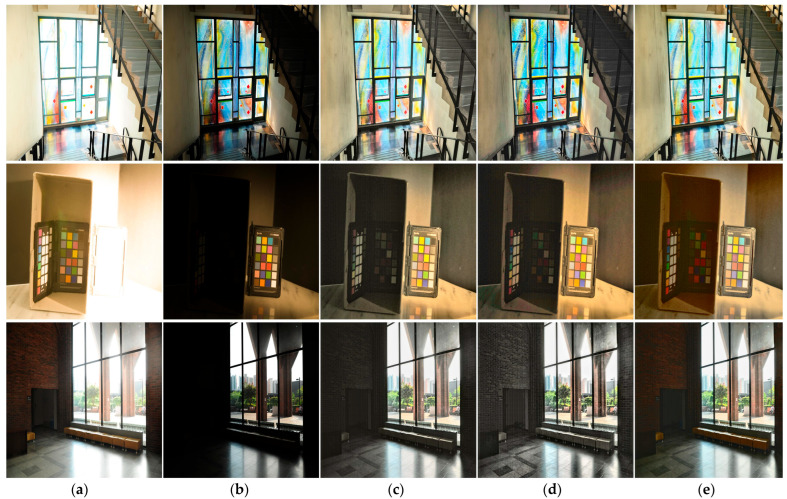
Results of the proposed inference process: (**a**) HE image; (**b**) LE image; (**c**) enhanced LE image; (**d**) color-compensated LE image; and (**e**) fused result.

To reduce color distortions introduced during enhancement, a luminance-based color compensation method is applied using the luminance difference between the original and enhanced LE images [34]. This approach mitigates color shifts caused by the enhancement process and preserves color consistency across varying luminance conditions:

(16) Lgain=LoutLin

(17) $${CC}_{gain}=\alpha \times L_{gain}^{\beta },\;\alpha =1.009,\;\beta =0.705$$

(18) $$a_{out}=\left(a_{in}-128\right)\times {CC}_{gain}+128$$

(19) $$b_{out}=\left(b_{in}-128\right)\times {CC}_{gain}+128$$

where α and β denote empirically determined parameters obtained from an experimental trend line. In the Lab color space, 128 corresponds to the neutral achromatic center. Therefore, each chromatic channel is normalized relative to this reference value, adjusted using ${CC}_{gain}$, and then shifted back to its original range. The resulting color-compensated LE image is fused with the original HE image and provided as input to the trained fusion module. This process reduces color distortion and improves fusion quality (Figure 8).

## 4. Experimental Results

This section presents ablation, qualitative, and quantitative evaluations of the proposed HE-LE fusion method. An ablation study is first conducted to examine the contribution of the proposed stage-wise loss optimization and preprocessing components. Qualitative comparisons across diverse exposure conditions are conducted to assess the ability of the proposed method to enhance visibility in underexposed regions while suppressing saturation and detail loss in overexposed areas. In addition, structural sharpness, color consistency, and tonal balance are examined to evaluate the visual naturalness of the fusion results. Quantitative evaluations using multiple image quality assessment metrics are further conducted to ensure an objective and fair comparison with existing methods.

For training, 4800 RAW images from the MIT-Adobe FiveK Dataset were used [35]. HE and LE images were generated from each RAW image by applying exposure compensations of +2 EV and –2 EV, respectively, to the same source image. This approach enabled the creation of training pairs free from temporal discrepancies or geometric misalignment. All experiments were conducted on a workstation equipped with an Intel Core i7-13700KF CPU, an NVIDIA GeForce RTX 4090 GPU with 24 GB of VRAM, and 64 GB of RAM. The proposed fusion module contains 1.904 M total parameters, all of which are trainable, and requires 404.328 GFLOPs per image pair. The weight file size of the fusion module is 7.38 MB. For the full inference pipeline, the HDR-like enhancement module required 289.995 ± 2.317 ms, while the color compensation step required 40.386 ± 7.982 ms. When combined with the fusion module inference time of 220.428 ± 0.730 ms, the total inference time of the full proposed pipeline was 550.809 ms per image, corresponding to 1.82 FPS. For evaluation, the test set consisted of the dataset provided by Lee et al. and several publicly available datasets widely used in previous studies [11,36,37,38]. These datasets were employed to assess the generalization capability of the proposed method across diverse scenes and illumination conditions.

The proposed method was compared with Mertens, DenseFuse, IFCNN, PMGI, MEFNet, U2Fusion, and TransMEF (Section 2). These methods include the traditional Multi-exposure Image Fusion approach, Mertens, CNN-based methods (DenseFuse, IFCNN, PMGI, MEFNet, and U2Fusion), and the transformer-based method TransMEF. This selection provides a comprehensive comparison with both conventional exposure fusion techniques and recent deep learning-based approaches.

For quantitative evaluation, non-reference image quality assessment (NIQA) metrics were adopted because HE-LE fusion does not provide ground-truth images, making full-reference metrics, such as PSNR and SSIM, unsuitable. Therefore, BRISQUE [39], NIQE [40], PIQE [41], SSEQ [42], CNNIQA [43], MANIQA [44], and JPEG2000 [45] were employed to assess image quality and naturalness. BRISQUE and NIQE measure distortions based on natural scene statistics, whereas PIQE and SSEQ evaluate perceptual quality and structural degradation. CNNIQA and MANIQA are deep learning-based metrics that better reflect human visual perception. In addition, JPEG2000 assesses image quality using spatial features, such as local contrast, structural distortion, and edge information, making it suitable for analyzing structural fidelity and detail preservation in the fused images.

Furthermore, entropy, average gradient (AG), and structural sharpness (SS) were used to evaluate information content and structural detail preservation. Entropy measures the amount of information contained in an image, while AG and SS quantify edge strength and structural sharpness. For BRISQUE, NIQE, PIQE, SSEQ, and CNNIQA, lower values indicate better performance. In contrast, higher values are preferred for MANIQA, Entropy, AG, SS, and JPEG2000, reflecting superior perceptual quality, information preservation, and structural fidelity.

### 4.1. Ablation Study

To verify the contribution of each component in the proposed method, a stepwise ablation study was conducted. The ablation study was designed to evaluate two aspects: the effect of the staged loss optimization schedule during training and the effect of the proposed preprocessing components during inference. For the training-stage analysis, four configurations were trained using 4800 images from the MIT-Adobe FiveK Dataset under the same total training length of 70 k iterations. These configurations compare different combinations of loss optimization stages. For the inference-stage analysis, the model trained with the final loss optimization schedule was used as the baseline, and HDR-like enhancement and color compensation were progressively applied during inference. As summarized in Table 2, Config. 1–Config. 4 evaluate the loss optimization schedule, whereas Config. 5 and Config. 6 evaluate the additional contribution of the proposed inference preprocessing components.

**Figure 9:**
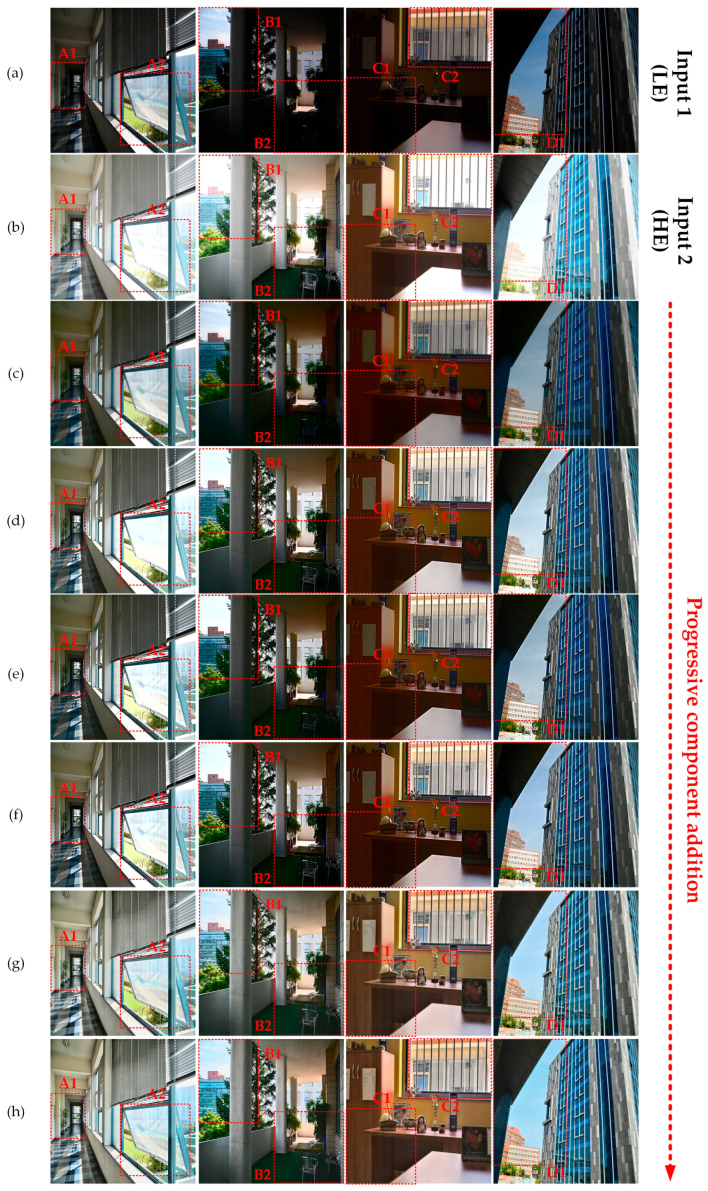
Representative visual comparison of the stepwise ablation configuration: (**a**) Low-Exposure input; (**b**) High-Exposure Input; (**c**) Config. 1; (**d**) Config. 2; (**e**) Config. 3; (**f**) Config. 4; (**g**) Config. 5; (**h**) Config. 6. The arrow indicates the progressive addition of loss optimization stages and inference preprocessing components. The labeled regions indicate representative exposure-dependent areas: A1, B2, and C1 correspond to dark regions where information is mainly preserved in the HE images, whereas A2, B1, and C2 correspond to bright regions where information is mainly preserved in the LE images. D1 indicates a region containing both bright and dark areas.

Figure 9 shows representative qualitative comparison results of the stepwise ablation study. Each result is compared using the same scenes and the same regions of interest, and the red dotted boxes indicate regions in which different information is preserved in the low-exposure and high-exposure images. First, when comparing Figure 9a,b, the low-exposure input in Figure 9a preserves relatively detailed information in bright regions, such as A2, whereas structural information in dark regions, such as A1, is barely visible. In contrast, the high-exposure input in Figure 9b improves the visibility of dark regions, but saturation and detail loss occur in bright regions. These complementary characteristics are also observed in the B1/B2, C1/C2, and D1 regions. Therefore, in HE-LE fusion, it is important to selectively utilize the effective regions from the two input images.

Figure 9c shows the result of the Config. 1, in which only Stage 1 loss optimization was applied. This stage was designed to allow the network to stably obtain the overall structure and basic luminance information from the two inputs, rather than to precisely select detailed information from the low-exposure and high-exposure images. As a result, information from both the low-exposure and high-exposure images is partially reflected; however, the sharpness of fine structures and local contrast are not sufficiently restored, resulting in an overall blurred appearance with insufficient detail.

Figure 9d shows the result of the Config. 2, in which the model was trained using the Stage 1 loss during the initial 40 k iterations and then switched to the Stage 2 loss for the remaining 30 k iterations. Compared with Figure 9c, boundaries and structural details are more clearly restored, and the sharpness of edge regions is particularly improved. However, in bright regions such as A2, C2, and D1, saturation artifacts caused by excessive enhancement are observed. This indicates that the tonal balance of bright regions can become unstable when structural sharpness is strengthened.

To alleviate this problem, Stage 3 loss optimization was added in the Config. 3. In Stage 3, the loss weight used in Stage 1, which strongly preserves the overall structure, was reduced, and an additional loss term was introduced to suppress over-saturation. However, because this stage can rapidly change the learning direction of the network, it was applied only for a short number of iterations during the overall training process and then switched back to Stage 2. As shown in Figure 9e, over-saturation is partially reduced, but the direction for selectively extracting effective information from the low-exposure and high-exposure images remains unclear. In other words, when the network is trained to refer to the low-exposure image for bright regions, it can also be affected by the bright regions of the high-exposure image. Conversely, when the network is encouraged to refer to the high-exposure image for dark regions, information from dark regions where the low-exposure image contains little useful information can also be reflected. These results indicate that a trade-off exists in the information selection process between the HE and LE inputs.

Figure 9f shows the result of the Config. 4, in which Stage 4 loss optimization was added. Stage 4 was designed to explicitly guide the reference direction based on the low-exposure image. Specifically, dark regions in the low-exposure image are encouraged to refer to the information in the high-exposure image, whereas bright regions in the low-exposure image are constrained to preserve information from the low-exposure image. This allows the network to move away from the previous learning strategy, in which the two input images were referenced in the same manner, and to adjust the region-wise reference relationship according to the exposure condition of the low-exposure image. As a result, Config. 4 alleviates the trade-off between over-saturation and detail loss and produces more balanced fusion results in both bright and dark regions.

In addition, the proposed network is designed to learn the selective utilization of effective information from the HE and LE inputs through the Transformer block inside the Deep Feature block. Therefore, even when HDR-like enhancement and color compensation preprocessing results, which have input distributions different from those used during training, are added during inference, the network can operate stably. Comparing Figure 9f,g, Config. 5 maintains the overall fusion structure even though HDR-like enhancement is applied to the low-exposure input and the input distribution is changed. In addition, the visibility and details of low-exposure regions are improved, but color fading is observed in some regions. Finally, in the Config. 6 result shown in Figure 9h, the addition of color compensation reduces this color distortion and improves overall tonal consistency and visual naturalness. Therefore, the stepwise comparison in Figure 9 qualitatively demonstrates that the proposed loss optimization strategy and inference-stage preprocessing components each contribute to improving the final fusion quality.

### 4.2. Qualitative Evaluation

In the qualitative evaluation, the proposed method is assessed from multiple visual perspectives, with particular emphasis on structural sharpness, color consistency, and tonal balance. Furthermore, its ability to enhance visibility in underexposed regions while suppressing saturation and detail loss in overexposed regions is examined.

The following figures present representative results for qualitative evaluation. Through these examples, the proposed method is visually compared with existing approaches, and its advantages are analyzed in detail based on selected regions of interest (ROI).

**Figure 10:**
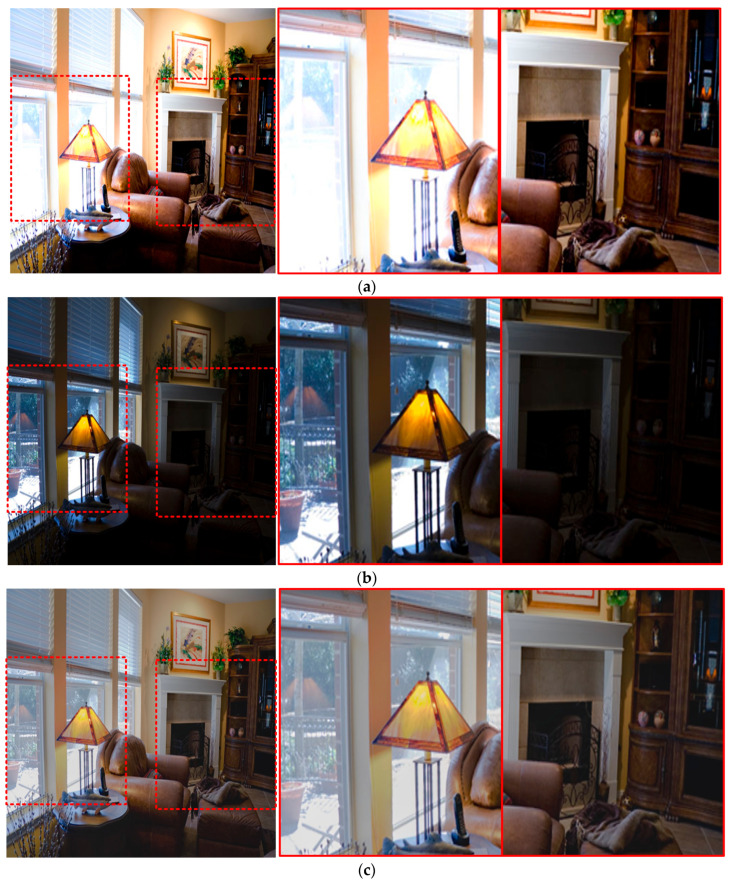

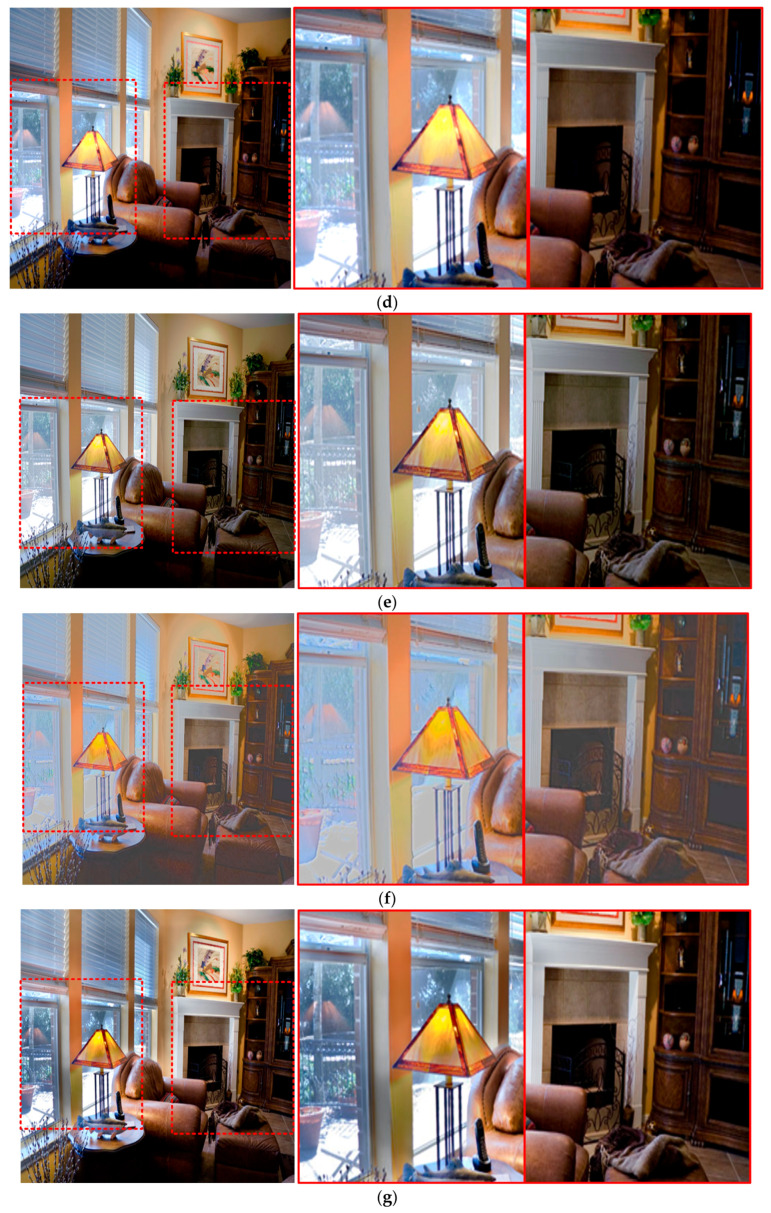

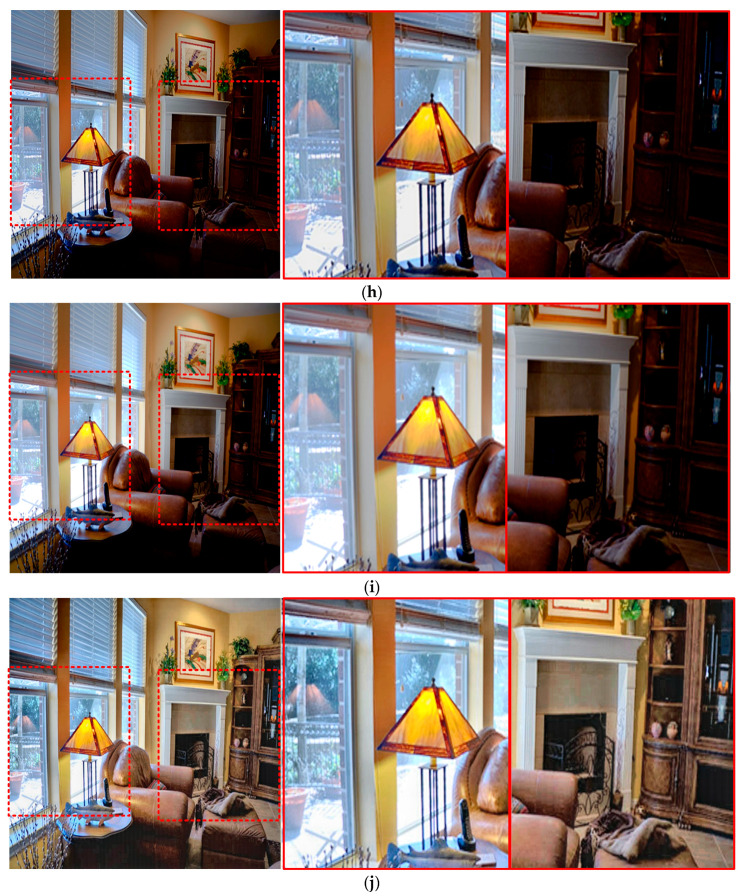
Qualitative comparison in a backlit indoor scene: (**a**) High Exposure Input; (**b**) Low Exposure Input; (**c**) Mertens; (**d**) DenseFuse; (**e**) IFCNN; (**f**) PMGI; (**g**) MEFNet; (**h**) U2Fusion; (**i**) TransMEF; (**j**) Proposed.

Figure 10 presents the qualitative comparison between the proposed method and existing Multi-exposure Image Fusion approaches. Mertens and PMGI exhibit noticeable highlight saturation around the window region, leading to partial loss of background details outside the scene. DenseFuse maintains a balanced overall luminance but shows reduced contrast in the fireplace and furniture regions on the right. IFCNN and MEFNet preserve indoor structural information relatively well; however, clipping artifacts remain visible in the bright areas surrounding the window.

In contrast, the proposed method preserves fine details of the trees and exterior structures visible through the window while enhancing visibility in dark indoor regions. Furthermore, contrast and color fidelity around the stand lamp are more naturally maintained, indicating a better balance between highlight preservation and shadow restoration.

**Figure 11:**
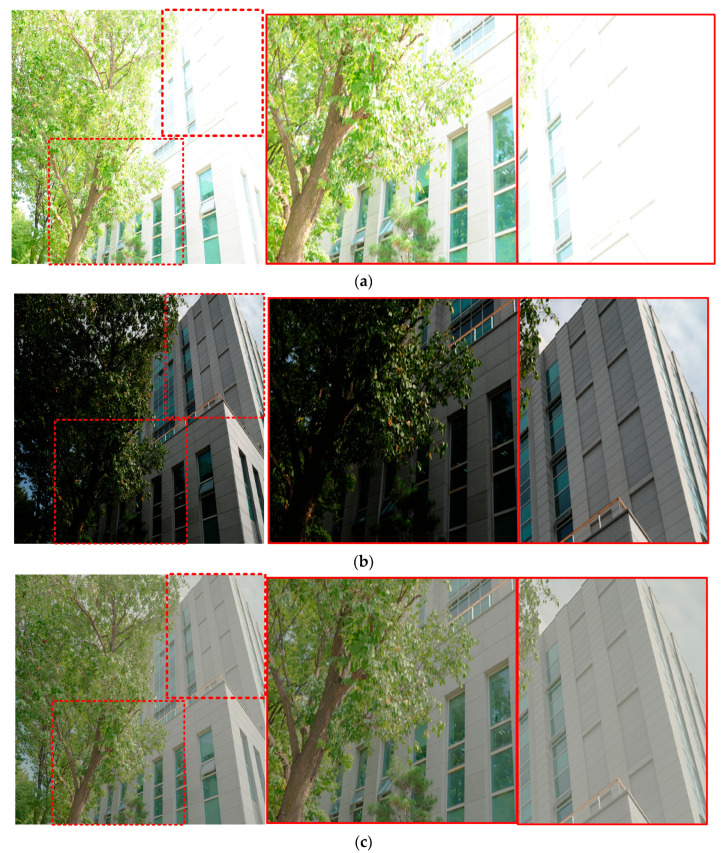

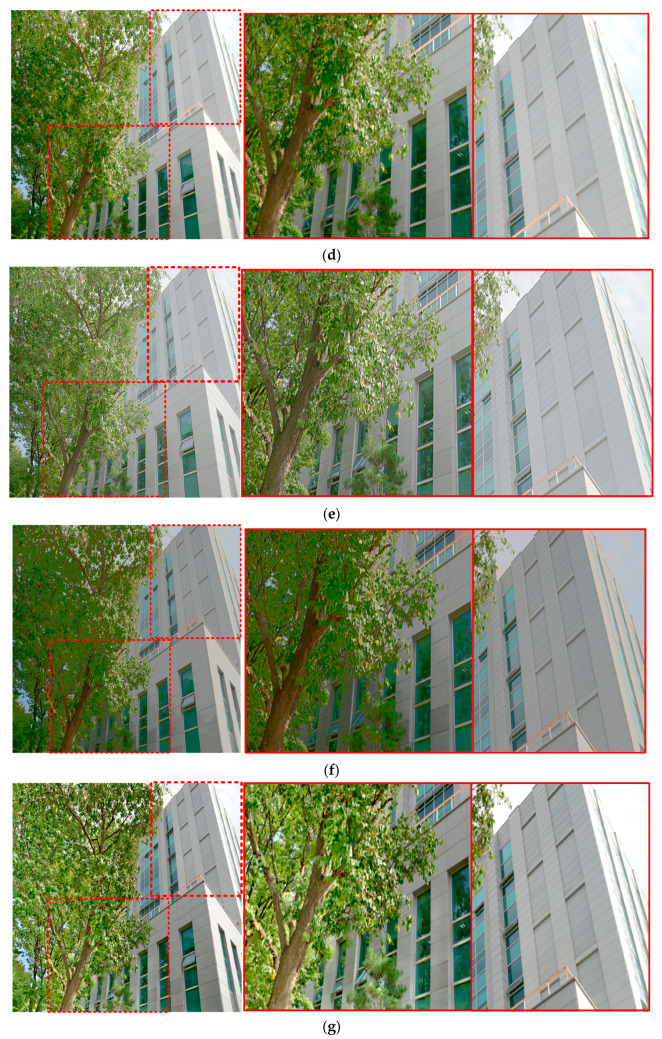

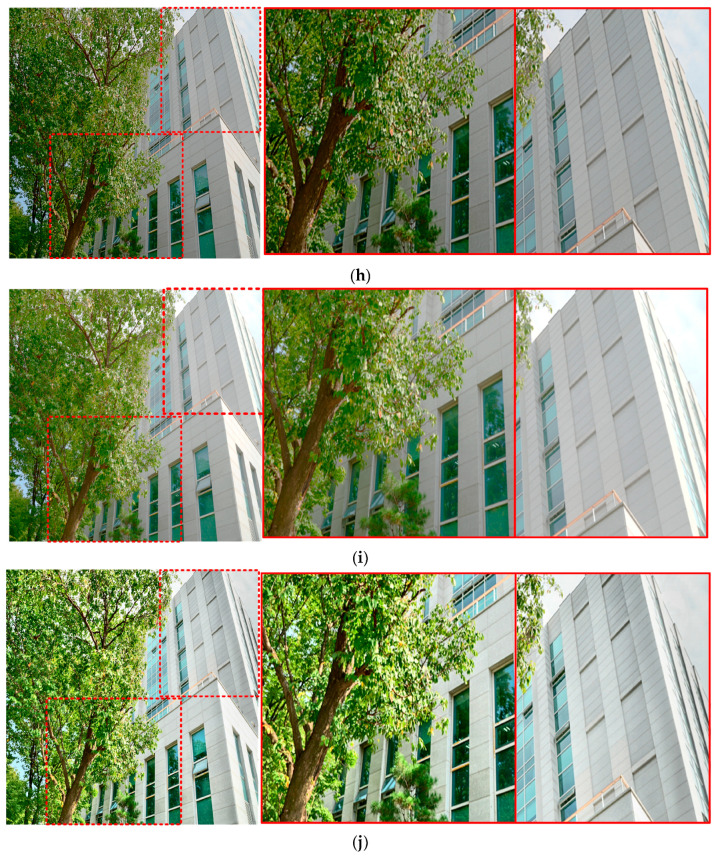
Qualitative comparison in a high-contrast outdoor scene. Enlarged views highlight texture and structural detail preservation: (**a**) High Exposure Input; (**b**) Low Exposure Input; (**c**) Mertens; (**d**) DenseFuse; (**e**) IFCNN; (**f**) PMGI; (**g**) MEFNet; (**h**) U2Fusion; (**i**) TransMEF; (**j**) Proposed.

Figure 11 presents an outdoor scene containing strong sunlight and complex foliage textures. A key evaluation criterion in this case is the ability to preserve structural details in the brightly illuminated building facade and foliage while maintaining color balance and contrast without excessive brightness enhancement. Mertens preserves overall luminance balance but exhibits reduced contrast at the building-sky boundaries, and fine foliage textures appear blurred. DenseFuse and IFCNN retain structural information relatively well; however, their outputs appear visually flat, reducing contrast and depth perception in the tree regions. PMGI reconstructs boundary structures clearly but introduces a noticeable green color bias across the image. MEFNet and U2Fusion provide satisfactory restoration of shadow regions and structural details, yet highlight that saturation and contrast imbalance remain visible around the windows and brightly illuminated façade areas.

In contrast, the proposed method effectively preserves highlight details in the windows and brightly illuminated façade regions while achieving sharper reconstruction of foliage textures and boundary structures. As highlighted by the red dashed box, it simultaneously retains the fine patterns of the building facade and the structural details of the trees without introducing excessive brightness enhancement or color distortion. Consequently, the proposed method produces more natural contrast and superior overall color balance than the competing approaches.

**Figure 12:**
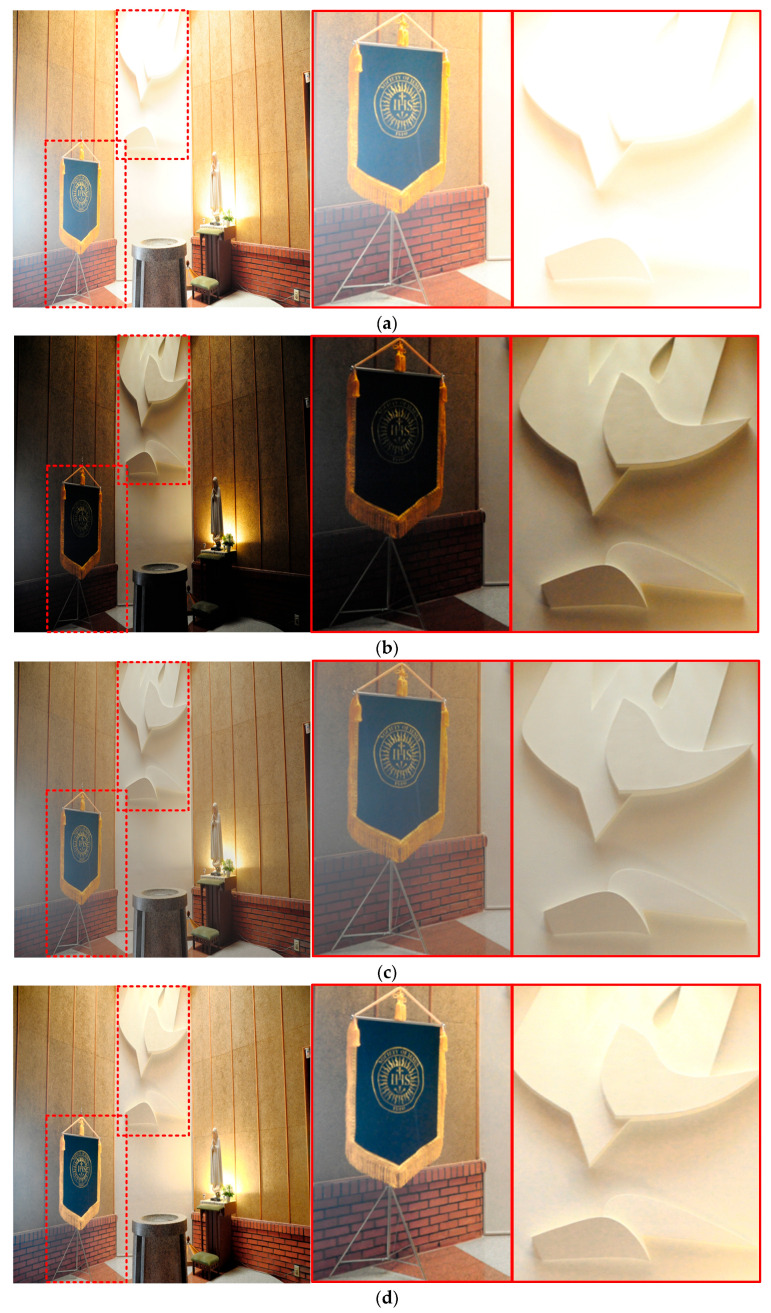

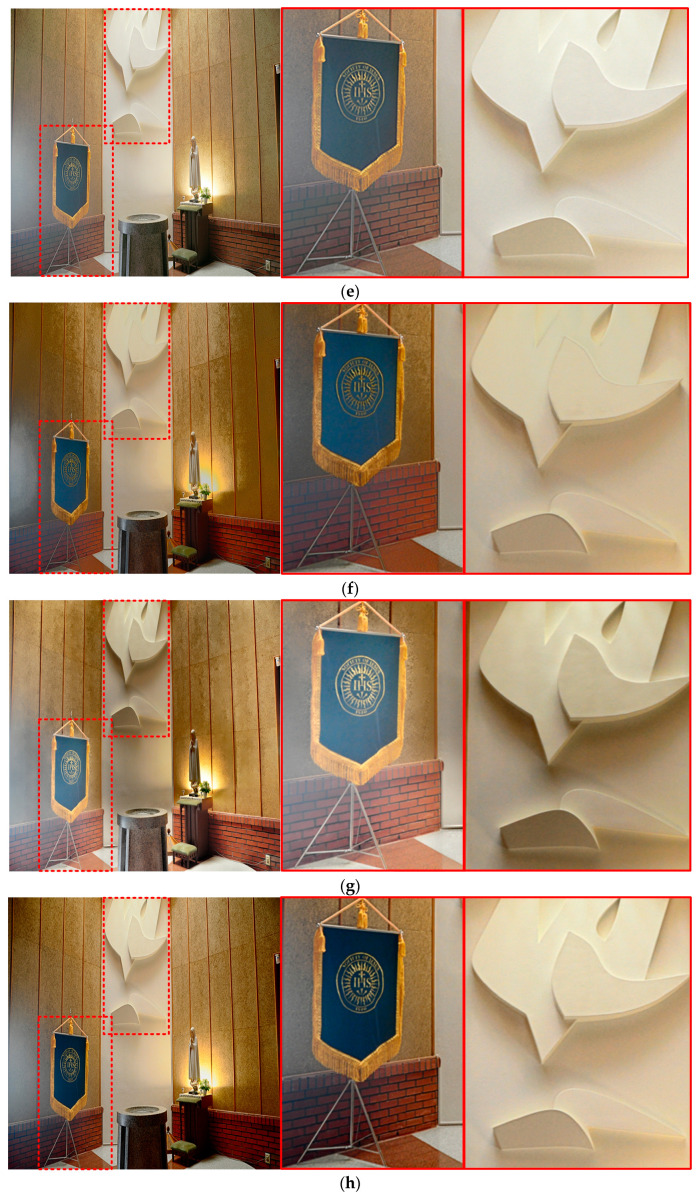

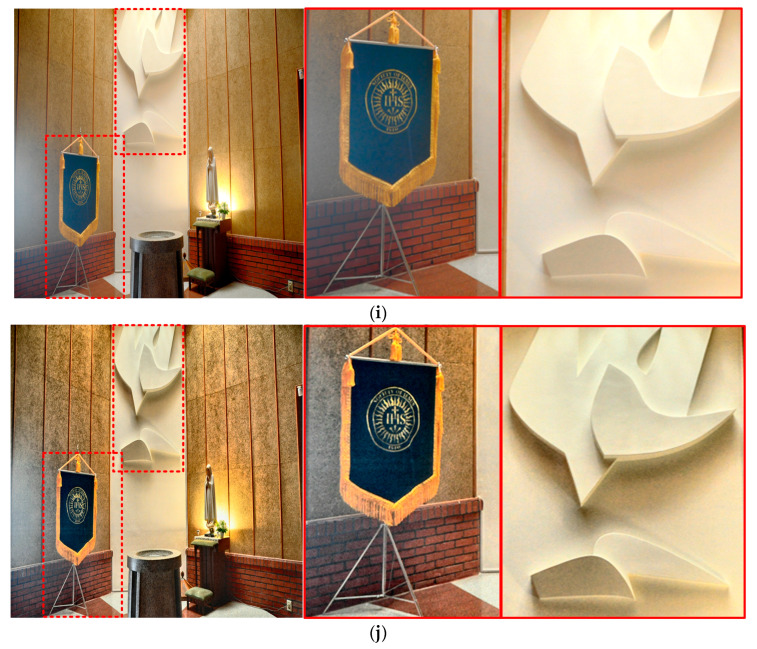
Qualitative comparison of texture and structural detail preservation in an indoor scene: (**a**) High Exposure Input; (**b**) Low Exposure Input; (**c**) Mertens; (**d**) DenseFuse; (**e**) IFCNN; (**f**) PMGI; (**g**) MEFNet; (**h**) U2Fusion; (**i**) TransMEF; (**j**) Proposed.

Figure 12 presents a scene containing localized illumination and bright wall structures. Mertens and DenseFuse enhance overall brightness but introduce excessive luminance amplification in the wall region on the right, causing partial loss of surface textures and shading details. IFCNN and PMGI preserve structures around the flag and wall regions well; however, their outputs appear visually flat, resulting in reduced local contrast and a weaker perception of surface geometry. MEFNet and U2Fusion improve visibility in dark areas, yet highlight saturation remains evident around the light source and bright wall regions, leading to diminished texture representation.

In contrast, the proposed method preserves highlight details in illuminated regions without excessive saturation while more accurately reconstructing the curved wall structures and shading variations on the right side. As highlighted in the red dashed box, it simultaneously retains the boundary details of the flag and the fine textures of the wall surface while maintaining stable luminance balance and contrast. Consequently, the proposed method produces a more visually natural fusion result than the competing methods.

**Figure 13:**
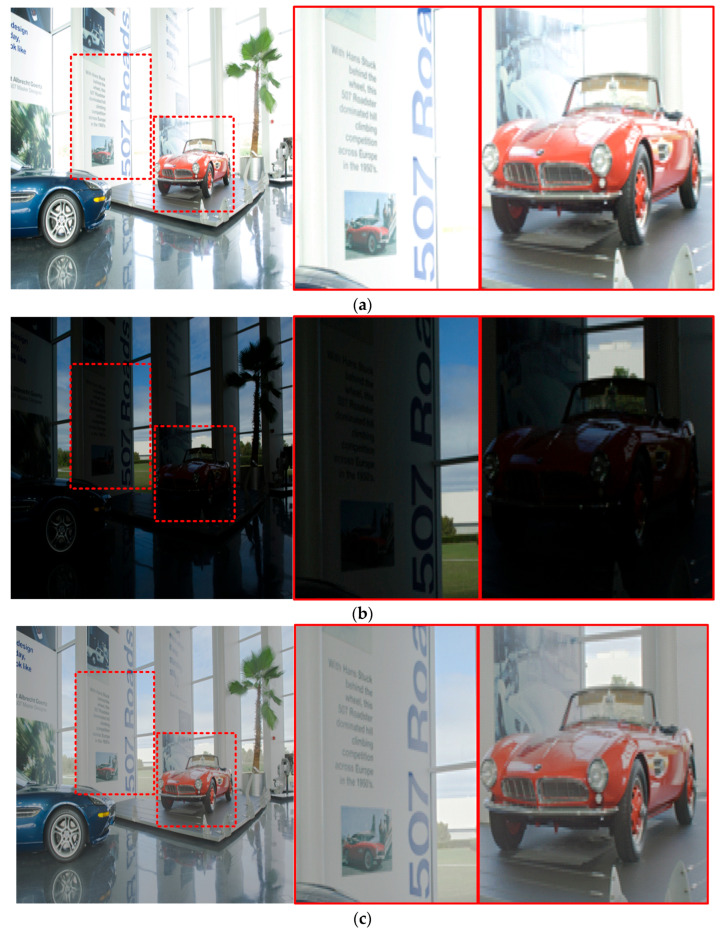

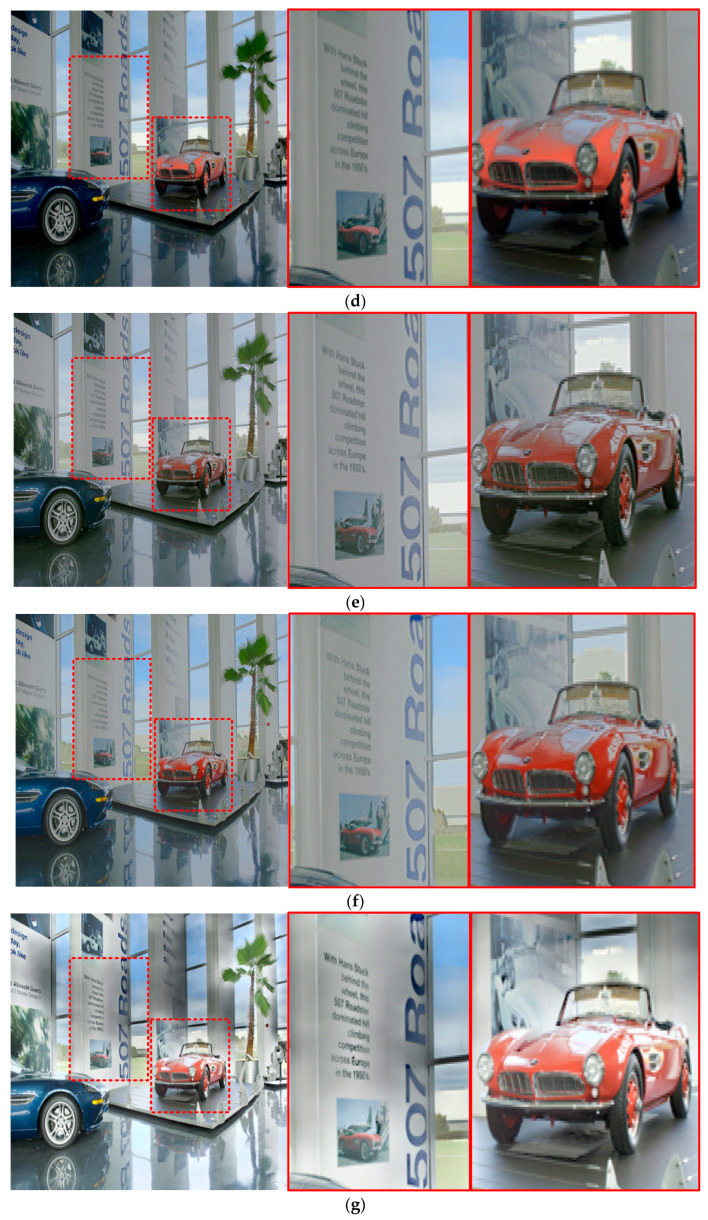

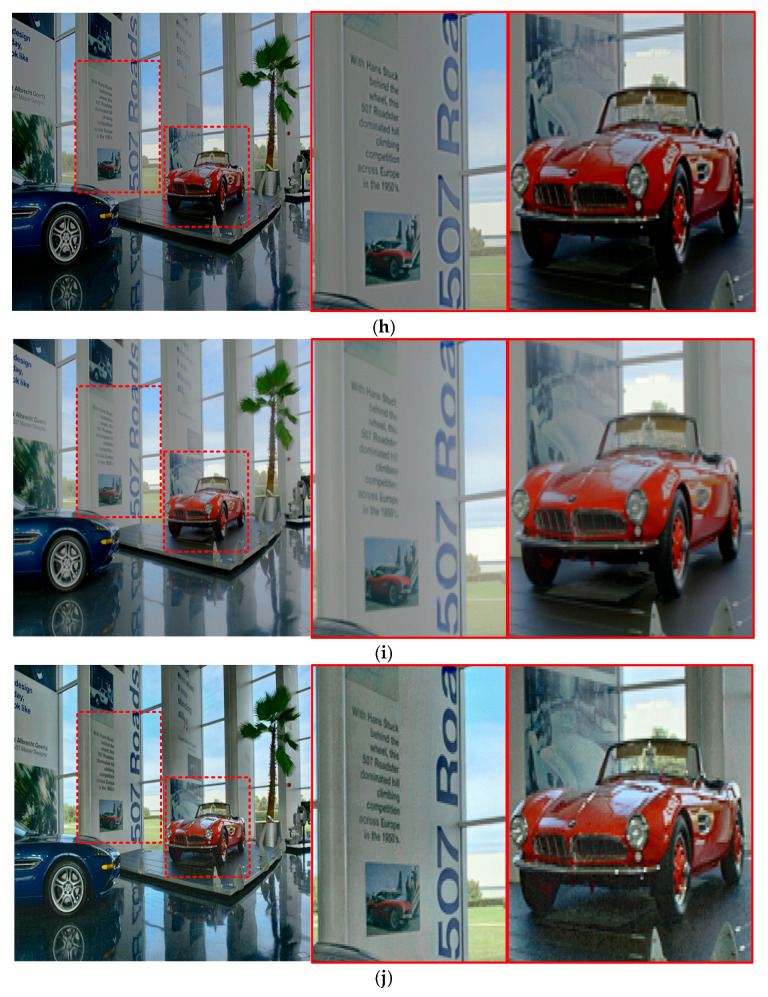
Qualitative comparison of text and structural detail preservation in an indoor scene: (**a**) High Exposure Input; (**b**) Low Exposure Input; (**c**) Mertens; (**d**) DenseFuse; (**e**) IFCNN; (**f**) PMGI; (**g**) MEFNet; (**h**) U2Fusion; (**i**) TransMEF; (**j**) Proposed.

Figure 13 presents an indoor exhibition scene with strong reflective effects. A key evaluation criterion in this case is the ability to simultaneously preserve vehicle-surface reflections and bright window regions naturally. Mertens maintains overall luminance balance but exhibits reduced contrast around the vehicle and floor reflections, causing fine textures and reflection details to appear blurred. DenseFuse and IFCNN preserve vehicle structures and indoor luminance information relatively well; however, their outputs appear visually flat, reducing depth perception in the front portion of the vehicle and the reflected floor regions. PMGI and U2Fusion retain vehicle color and structural details more effectively, but highlight saturation remains evident in the bright window areas surrounding the vehicle, accompanied by local contrast imbalance.

In contrast, the proposed method more effectively preserves the textures of the vehicle front and the reflection details on the vehicle body while restoring brightness information around the windows without excessive saturation. As highlighted in the red dashed box, it simultaneously retains reflective structures on the vehicle and floor reflection details within the exhibition environment. Furthermore, vehicle color fidelity and contrast are more naturally restored, resulting in a more balanced and visually pleasing fusion result.

**Figure 14:**
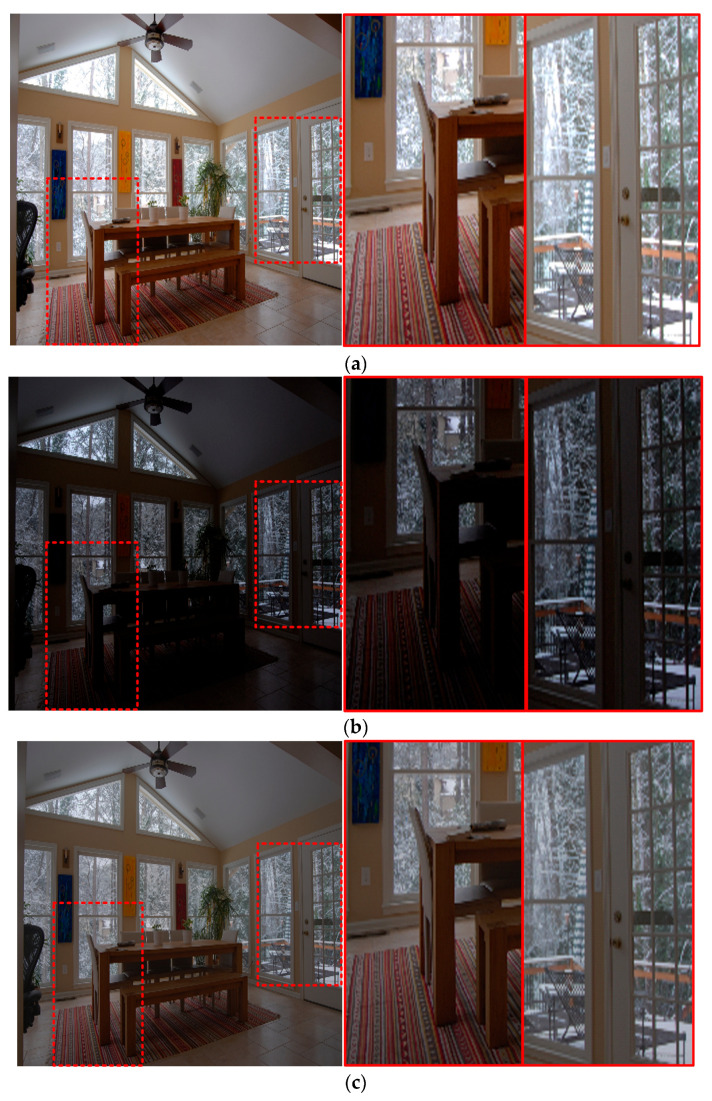

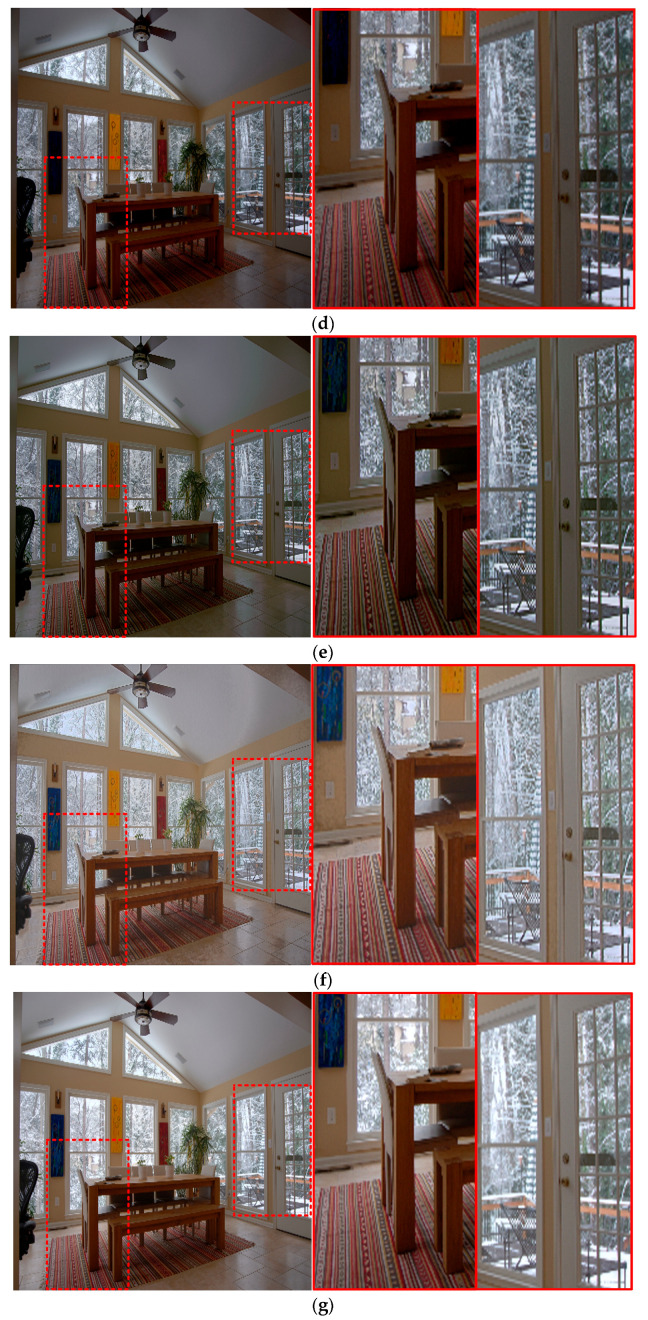

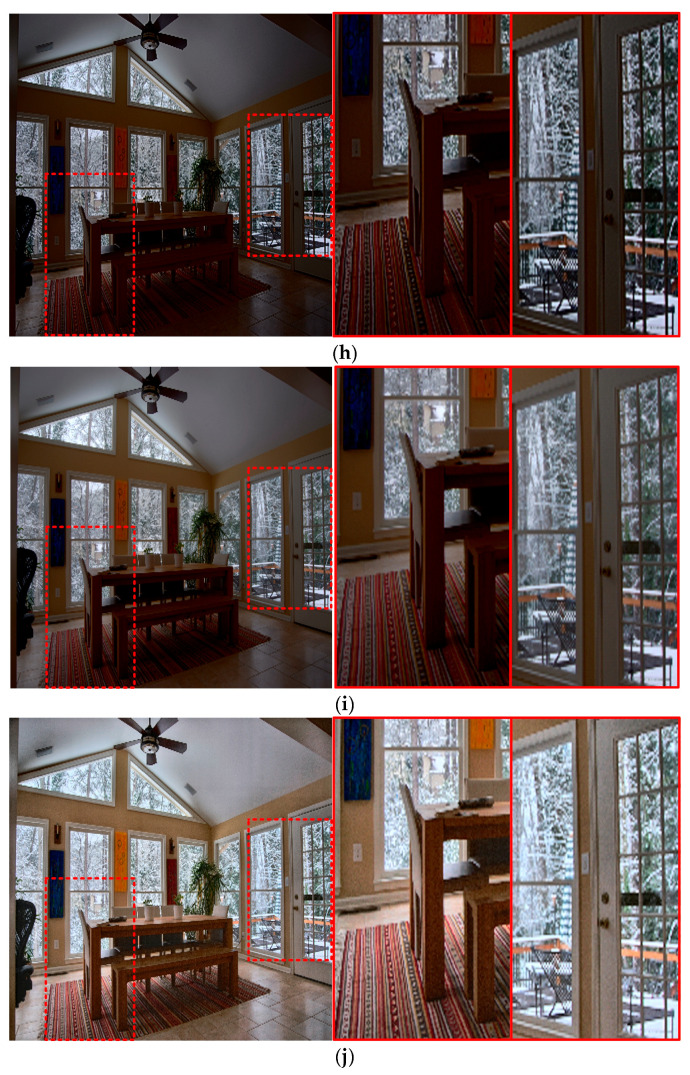
Qualitative comparison of highlight and shadow detail preservation in an indoor scene with bright outdoor illumination: (**a**) High Exposure Input; (**b**) Low Exposure Input; (**c**) Mertens; (**d**) DenseFuse; (**e**) IFCNN; (**f**) PMGI; (**g**) MEFNet; (**h**) U2Fusion; (**i**) TransMEF; (**j**) Proposed.

Figure 14 illustrates a scene containing dark indoor regions and snow-covered outdoor areas. In this case, effective fusion requires preserving the structural details of the snow-covered exterior while simultaneously enhancing visibility in indoor shadow regions. Mertens maintains a relatively balanced luminance distribution but exhibits reduced contrast around the bright window areas, causing outdoor snow textures and structural details to appear blurred. DenseFuse and PMGI provide satisfactory restoration of indoor brightness; however, excessive overall brightening reduces contrast around the table and interior walls, making window-frame boundaries appear visually flattened. U2Fusion and TransMEF preserve outdoor structural details relatively well but produce darker outputs, resulting in insufficient visibility beneath the table and in shadowed regions on the left. IFCNN and MEFNet achieve a more balanced indoor–outdoor luminance distribution, although fine snow textures outside the window and structural contrast within the room are partially weakened.

In contrast, the proposed method more effectively preserves the snow textures and tree structures outside the window while restoring details in the shadowed regions around the indoor table and chairs. As highlighted in the red dashed box, it naturally maintains the boundaries between the window frame and outdoor structures while achieving a balanced indoor-outdoor luminance distribution without excessive brightening or contrast flattening. In addition, structural details in the table legs and floor regions are reconstructed more clearly, resulting in a visually natural and well-balanced fusion output.

**Figure 15:**
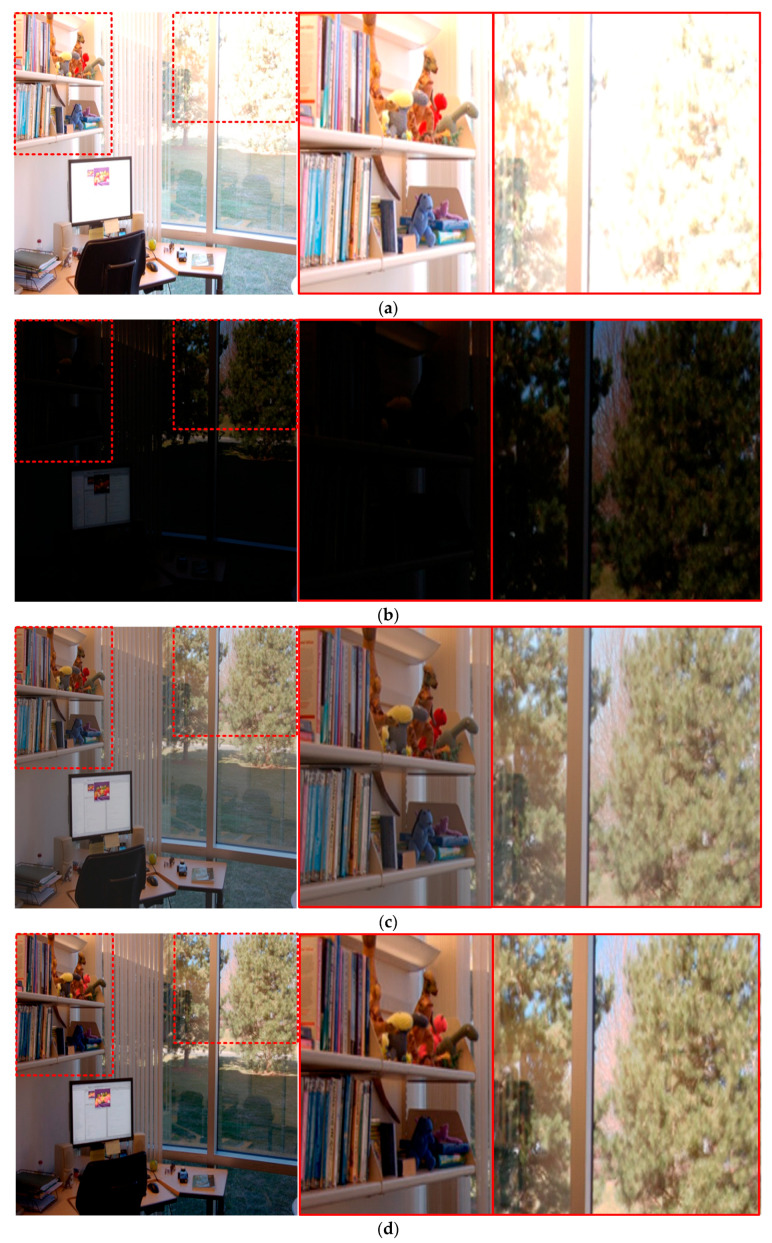

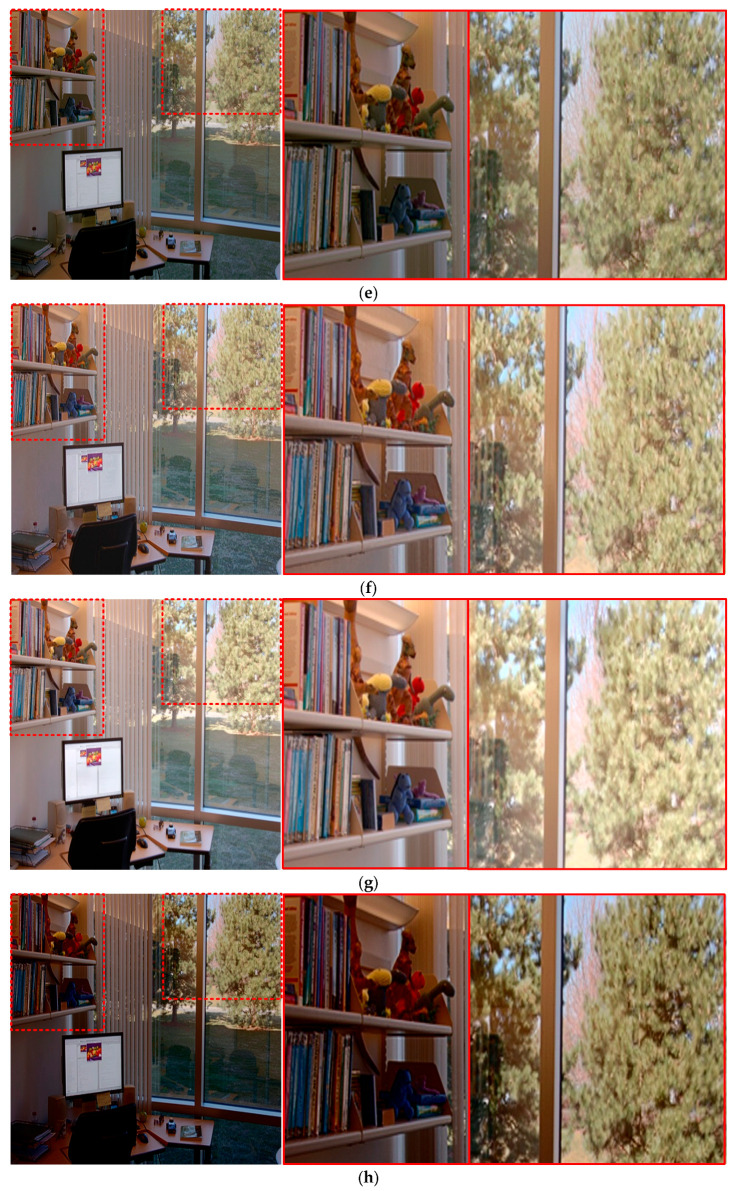

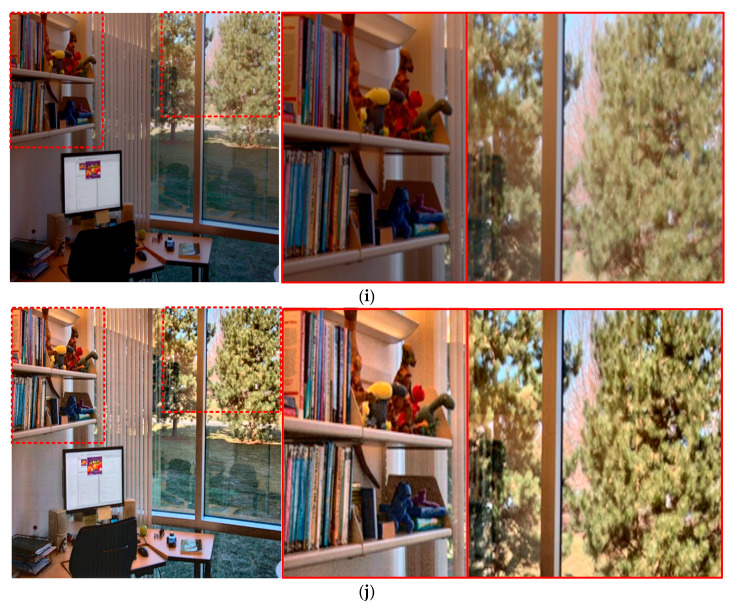
Qualitative comparison of indoor structural and outdoor texture preservation: (**a**) High Exposure Input; (**b**) Low Exposure Input; (**c**) Mertens; (**d**) DenseFuse; (**e**) IFCNN; (**f**) PMGI; (**g**) MEFNet; (**h**) U2Fusion; (**i**) TransMEF; (**j**) Proposed.

Figure 15 presents a scene with complex indoor textures and strong backlighting. Mertens maintains a relatively balanced luminance distribution; however, tree textures and boundary details outside the window appear blurred, and contrast within the bookshelf region is reduced. DenseFuse and IFCNN preserve indoor structures and outdoor brightness information reasonably well, but their outputs appear visually flat, leading to reduced contrast near the windows and weaker texture representation inside the bookshelf. PMGI and U2Fusion retain outdoor tree structures more effectively, yet produce darker images with reduced visibility beneath the bookshelf and around the desk. MEFNet and TransMEF provide satisfactory brightness restoration, but partial saturation and contrast flattening occur around the bright window regions, resulting in the loss of fine structural details in the outdoor background.

In contrast, the proposed method more effectively preserves the textures and boundary details of the trees outside the window while restoring fine structural details in the bookshelf and desk regions. As highlighted in the red dashed box, it naturally maintains the color and texture representation of the bookshelf contents while enhancing window-region contrast without excessive brightness amplification. Furthermore, the method simultaneously preserves outdoor structural details and indoor shadow visibility, resulting in a more balanced and visually natural fusion result.

### 4.3. Quantitative Evaluation

The quantitative evaluation was conducted using NIQA metrics, as HE-LE fusion does not provide ground-truth reference images. To comprehensively assess image quality, seven NIQA metrics were employed: BRISQUE, NIQE, PIQE, SSEQ, CNNIQA, MANIQA, and a JPEG2000-based metric.

BRISQUE, NIQE, and PIQE are widely used NIQA metrics that assess image distortion based on natural scene statistics, whereas SSEQ evaluates quality by jointly considering structural and spectral information. CNNIQA and MANIQA are deep learning-based metrics trained on large-scale datasets to better reflect human perceptual judgments. In addition, the JPEG2000-based metric indirectly assesses structural fidelity and image complexity through compression efficiency characteristics.

However, NIQA metrics are black-box measures whose decision processes are difficult to interpret and may be biased toward specific distortion types. To complement these limitations, low-level statistical metrics, including entropy, AG, and SS, were additionally employed to assess information content, edge strength, and SS.

To ensure a fair assessment of generalization performance, quantitative evaluations were conducted on 100 randomly selected images. The evaluation set included not only images from the MIT-Adobe FiveK dataset used for training, but also publicly available datasets excluded from the training process. This protocol reduces dataset-specific bias and enables evaluation across diverse scenes and imaging conditions.

**Figure 16:**
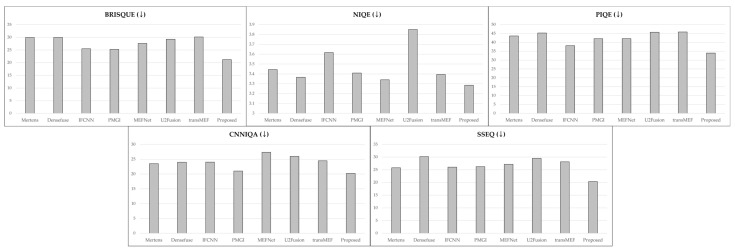
Comparison of BRISQUE, NIQE, PIQE, SSEQ, and CNNIQA scores (↓ indicates that lower values are better).

**Figure 17:**
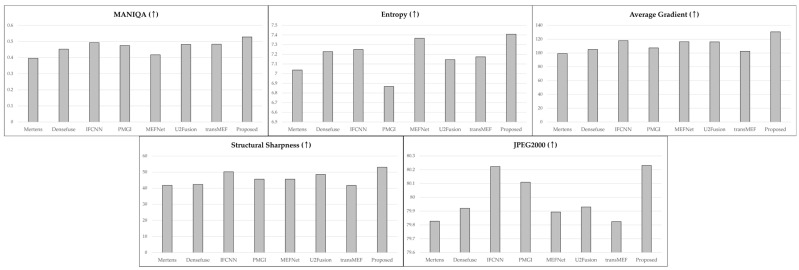
Comparison of MANIQA, Entropy, AG, SS, and JPEG2000 scores (↑ indicates that higher values are better).

Table 3 summarizes the average results for all evaluation metrics. The arrows indicate the preferred performance direction, where ↓ denotes lower-is-better and ↑ denotes higher-is-better. Figure 16 and Figure 17 visualize the metrics favoring lower and higher values, respectively, using bar charts. As shown, the proposed method consistently achieves superior performance across the majority of evaluation metrics.

### 4.4. Verification on Real-Captured Multi-Exposure Samples

**Figure 18:**
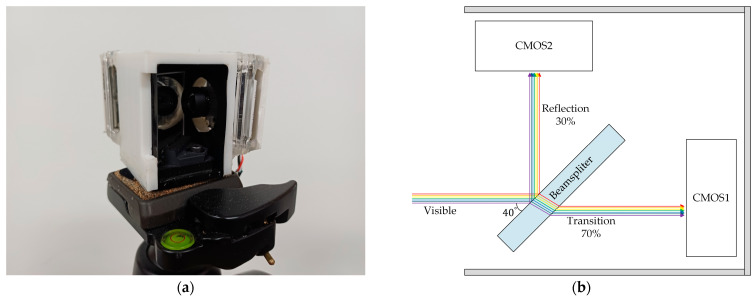
Imaging setup used for real-captured multi-exposure sample acquisition: (**a**) Camera configuration; (**b**) top-view layout of the acquisition setup.

To further verify the practical applicability of the proposed method, we conducted an additional experiment using real-captured multi-exposure samples obtained with our imaging setup. Figure 18 shows the imaging setup used to acquire the real-captured HE and LE images. The setup follows the system configuration described in [46], where the detailed hardware specifications are also presented. In this study, the same setup was used to capture visible images under different exposure settings from real scenes.

The proposed network is trained using virtual HE–LE pairs generated from a single RAW image, which provides pixel-level aligned training data without misregistration errors. However, this training strategy does not restrict the inference input to RAW-synthesized pairs. During inference, the proposed method can be applied to HE and LE images obtained from multi-exposure inputs.

**Figure 19:**
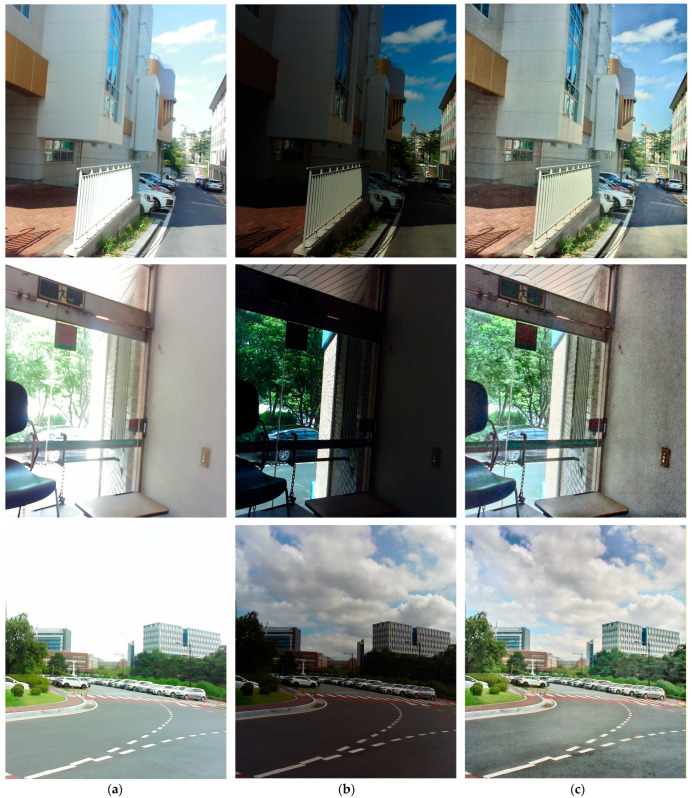
Verification results using samples acquired with the real-captured imaging setup: (**a**) high-exposure image, (**b**) low-exposure image, and (**c**) fused result obtained by the proposed method.

Figure 19 shows the verification results obtained from the real-captured HE and LE samples. This experiment is intended as an additional real-captured sample verification, rather than a replacement for the comparative benchmark evaluation presented in Section 4.2 and Section 4.3. As shown in Figure 19, the proposed method successfully generates fused images from physically captured HE and LE inputs. The fused results improve visibility in underexposed regions while preserving details in bright regions. They also maintain stable color appearance and structural consistency, indicating that the proposed fusion pipeline can be applied to real-captured multi-exposure inputs beyond the RAW-synthesized training pairs.

## 5. Conclusions

Single-image dynamic range expansion methods avoid alignment and dataset construction challenges, but their ability to recover missing information is limited by inference-based reconstruction. In contrast, HE-LE fusion can exploit complementary information from multiple exposures, although its performance is often affected by alignment errors and dataset construction difficulties.

To address these limitations, this study proposed an alignment-free dataset generation strategy that creates HE-LE pairs with different exposure ranges from a single 14-bit RAW image. By generating fully aligned training pairs from a common source, the proposed approach mitigates the dataset dependency and alignment issues in conventional fusion-based learning frameworks.

Furthermore, inspired by cross-band fusion architectures, the proposed framework separately processes shallow and deep features to capture complementary information at different representation levels. Recognizing that the relationship between HE and LE images is spatially varying and non-fixed, an adaptive fusion strategy was developed to effectively model their complementary characteristics. In addition, a stage-wise loss optimization was introduced to stabilize training and promote balanced integration of information across different exposure levels.

During inference, HDR-like enhancement was applied as a pre-processing step to compensate for luminance and color deficiencies in the LE image. The enhanced LE image was then refined through L-gain-based color compensation and fused with the HE image to generate the final output.

Both qualitative and quantitative results demonstrated that the proposed method consistently outperforms existing approaches across diverse imaging conditions. In particular, it effectively enhances visibility in underexposed regions while suppressing saturation in overexposed regions. Moreover, the proposed learning strategy enables stable training without HDR ground truth and simultaneously mitigates dataset construction and alignment challenges in HE-LE fusion.

Future research may focus on improving generalization across diverse imaging environments and sensor types, as well as extending the framework to real-time applications and multi-modal fusion tasks. The proposed RAW-based dataset generation strategy and learning framework may also apply to a broader range of image enhancement and fusion problems.

Overall, this study addresses key limitations of HE-LE fusion-based image enhancement and introduces a robust and effective framework for both training and inference.

## Figures and Tables

**Table 1:** Loss-weights for stage-wise loss optimization.

Loss	Stage 1	Stage 2	Stage 3	Stage 4
$L_{ms\_ssim}$	30	30	5	5
$L_{int}$	1	1	0.5	0.5
$L_{grad}$	1	1	2	2
$L_{lap}$	0	1	2	2
$L_{clip}$	0	0	0.1	0
$L_{exp}$	0	0	0	1

**Table 2:** Configurations of the ablation study.

Configuration	Training Loss Optimization Schedule	Inference Preprocessing
Config. 1	Stage 1: 70 k	None
Config. 2	Stage 1: 40 k + Stage 2: 30 k	None
Config. 3	Stage 1: 30 k + Stage 2: 20 k + Stage 3: 5 k + Stage 2: 15 k	None
Config. 4	Stage 1: 30 k + Stage 2: 20 k + Stage 3: 5 k + Stage 4: 15 k	None
Config. 5	Same as C4	HDR-like Enhancement
Config. 6	Same as C4	HDR-like Enhancement + Color Compensation

**Table 3:** Quantitative comparison of IQA metrics on 100 test images.

Name/Metric	Mertens	Densefuse	IFCNN	PMGI	MEFNet	U2Fusion	TransMEF	Proposed
BRISQUE (↓)	29.938	29.986	25.520	25.283	27.624	29.271	30.155	21.214
NIQE (↓)	3.4443	3.3664	3.6169	3.4089	3.3406	3.8506	3.3941	3.2853
PIQE (↓)	43.592	45.286	38.114	42.056	42.085	45.734	45.871	33.979
SSEQ (↓)	25.803	30.213	26.021	26.249	27.165	29.507	28.136	20.367
CNNIQA (↓)	23.505	23.979	24.028	21.025	27.348	25.970	24.495	20.204
MANIQA (↑)	0.3958	0.4526	0.4926	0.4749	0.4175	0.4820	0.4835	0.5280
Entropy (↑)	7.0381	7.2284	7.2508	6.8665	7.3669	7.1452	7.1745	7.4091
AG (↑)	99.124	105.15	117.88	107.30	116.34	116.18	102.45	130.71
SS (↑)	41.778	42.350	50.158	45.585	45.584	48.509	41.759	53.016
JPEG2000 (↑)	79.827	79.920	80.223	80.110	79.894	79.930	79.824	80.231

AG: Average-Gradient, SS: Structural-Sharpness. Note: ↑ indicates that higher values are better, whereas ↓ indicates that lower values are better.

## Data Availability

The datasets used in this study are publicly available from their original sources. Additional data generated during the study are available from the corresponding author upon reasonable request.
